# “I just can’t do that anymore”: a qualitative exploration of symptoms and function in patients living with abdominal wall hernia (AWH)

**DOI:** 10.1007/s10029-025-03489-3

**Published:** 2025-10-14

**Authors:** Asim Ahmad Abbas, Olivia A. Smith, Mark Mierzwinski, Thomas MacLeod, Praminthra Chitsabesan, Srinivas Chintapatla

**Affiliations:** 1 York Abdominal Wall Unit, Department of General Surgery, 4th Floor, Administrative Block, York Hospital, York & Scarborough Teaching Hospitals NHSFT, Wigginton Road, Clifton, YO31 8HE York, UK; 2 School of Science, Technology and Health, York St John University, Lord Mayor’s Walk, York, UK

**Keywords:** Abdominal wall hernia, Qualitative, Phenomenology, Symptoms, Function, Biopsychosocial, Chronic, Restriction, Adaptation

## Abstract

**Background:**

Abdominal Wall Hernia (AWH) is a structurally and functionally disruptive condition often assessed through objective clinical variables such as position, size, reducibility, skin changes, and symptoms of obstruction suggesting the need for urgent treatment. However, little attention has been given to explore all the symptoms experienced. Many Health-Related Quality of Life (HRQoL) tools fail to capture the nuances of lived experience, especially around pain, restriction, and behavioural response. This study provides a qualitative examination of the symptomatology and functional impact of AWH as described from the patient’s perspective.

**Methods:**

This phenomenological study draws on 15 semi-structured interviews with patients living with AWH. Participants were sampled purposively across a spectrum of hernia complexity (VHWG grades 1–4). Transcripts were analysed using Interpretative Phenomenological Analysis (IPA) to generate superordinate and subordinate themes concerning symptoms and functional impact. Data analysis was triangulated between qualitative and surgical researchers.

**Results:**

The analysis revealed a superordinate theme of “Symptoms and Function,” with three distinct but overlapping subordinate domains: management of pain, freedom of movement, and restriction and adaptation. Participants described persistent background pain, episodic sharp exacerbations, and visceral sensations (e.g., bloating and urinary urgency). Anticipatory vigilance shaped movement planning, with participants choreographing basic tasks and progressively surrendering valued activities across both leisure and employment. Restriction triggered inventive adaptations, including custom abdominal binders and adjusted routines.

**Conclusions:**

AWH imposes a diffuse, dynamic and difficult burden on patients’ bodies and lives, extending far beyond the anatomical boundaries typically measured in clinical practice. Participants described a constellation of physical symptoms, including pain, visceral discomfort, and movement restriction. Our findings highlight that functional limitation in AWH is a biopsychosocial phenomenon. These experiences resonate with pain-psychology frameworks and demonstrate the limitations of reductionist surgical assessments. Effective interventions must match the complexity of lived experience, combining surgical, psychological, and rehabilitative strategies.

**Supplementary Information:**

The online version contains supplementary material available at 10.1007/s10029-025-03489-3.

## Background

Abdominal Wall Hernia (AWH) is a complex, often progressive condition that disrupts the structural and functional integrity of the abdominal wall [1]. It is therefore not surprising that in surgical practice AWH is often conceptualised as a biomechanical defect associated with bulging, pain, and risk of incarceration. However, the effect of AWH on patients’ lives is wide-reaching, causing significant functional impact [2]. Despite its broad impact, Health-Related Quality of Life (HRQoL) assessments in AWH often prioritise general or post-surgical functionality, such as recurrence and wound healing, while overlooking more nuanced aspects of lived experience [3]. For instance, current HRQoL tools may ask if a patient can “lift”, “sit up” or “walk” but rarely explore *how* and *why* such activities are modified, feared, or avoided, nor the wider ranging impact such limitations have for the patients [4–6]. These omissions risk underestimating the extent of functional limitation and the emotional toll of AWH.

Treatment for AWH aims to restore anatomical form and reduce the risk of life-threatening complications. Yet the full burden of symptoms and the extent to which function is compromised are seldom explored in depth during surgical assessment [7]. Neglect or omission may reflect a broader clinical tendency to interpret function through objective measures, such as hernia size or reducibility, rather than subjective experience. For example, the European Hernia Society (EHS) classification system, though widely adopted, is based primarily on anatomical variables and expert consensus, and recent multicentre data suggest that while hernia width and type predict some clinical outcomes, many patient-relevant dimensions remain uncaptured within this framework [8].

This study applies a phenomenological approach to examine how patients with AWH experience pain, movement restriction, and adaptation. This paper presents a detailed extension of the index study which identified five superordinate domains (Body Image [9], Symptoms and Function, Interpersonal Relationships [10], Employment, and Mental Health [11]) underpinning patients’ lived experiences of AWH [7]. By exploring the superordinate theme of “Symptoms and Function,” we aim to generate insight into how physical symptoms are felt, managed, and incorporated into daily life. We then consider if there are any implications for clinical practice from these insights. 

## Methods

### Study design

This study employed a phenomenological methodology to explore patients’ lived experiences of symptoms and functional impact arising from AWH. A total of fifteen semistructured interviews were conducted to explore how AWH altered participants’ relationship with their body, movement, pain, and daily functioning. Narratives were analysed using Interpretative Phenomenological Analysis (IPA). Triangulation of interpretations was undertaken collaboratively by two gastrointestinal surgeons (SC, PC) with specialist interest in abdominal wall reconstruction and a qualitative researcher (MM) to strengthen interpretive rigour.

### Ethics

Ethical approval was obtained through Hull York Medical School (HYMS), the Integrated Research Approval System (IRAS), and the UK Health Research Authority (HRA), under Research Ethics Committee reference 19/SC/0565. Written and verbal informed consent was obtained from all participants prior to participation. Declaration of Helsinki and the Consolidated criteria for REporting Qualitative (COREQ) guidelines were adhered to. Pseudonyms are used throughout this manuscript in accordance with ethical protocols and to honour the narrative integrity of participants’ accounts.

### Recruitment

Participants were recruited through purposive sampling from specialist abdominal wall clinics at a tertiary centre in the UK. This approach was chosen to identify “information-rich cases” that reflected a wide spectrum of symptomatic experience, including pre- and post-operative patients across all Ventral Hernia Working Group (VHWG) grades 1–4 [12]. Participants had a variety of comorbidities, including a history of colorectal cancer, stoma, smoking, presence of fistula, diabetes mellitus, obesity, and inflammatory bowel disease. While the VHWG system is primarily used to predict perioperative risk rather than symptom burden, it provided a pragmatic framework to capture variation in comorbidity and hernia characteristics. The sample included incisional, parastomal, and primary midline hernias, with both midline and lateral defects represented. Lumbar hernias were not present. Detailed participant demographics, including CT-defined hernia dimensions and comorbidities, are presented in Table 1 (Supplementary File 1). Participants received a letter of invitation with an attached participant information sheet. A more detailed overview of the recruitment methodology is available in supplementary project publications [7]. The number of participants was not predetermined; recruitment continued until thematic saturation was achieved.

The sample included patients with a range of abdominal wall hernia presentations, most of whom would be classified as “complex” based on width, recurrence, stoma presence, or prior mesh repair. This reflects the typical case-mix at our tertiary referral centre. However, participant inclusion was based on symptomatic impact rather than anatomical criteria alone. We deliberately did not restrict to or foreground the term “complex AWH” in our framing of this study, as our primary aim was to explore the lived experience of hernia-related symptoms, regardless of formal operative complexity.

### Data collection

Semi-structured interviews, supported by a topic guide (Supplementary File 2, topics a and e), were used to elicit narrative style responses and allow participants to direct the interview toward personally salient aspects of their experience. The topic guide was developed collaboratively by qualitative researchers and surgeons and included probing questions on pain, movement, physical function, and bodily adaptation. Interviews were intentionally broad in scope to allow for the emergence of unanticipated symptom descriptions and responses to restrictions and adaptations in function [13]. All interviews were conducted by OS (Research Fellow), who had no prior relationship with participants.

### Data analysis

Data was analysed thematically using IPA within NVivo v12 software (QSR International). IPA was chosen because it is specifically concerned with how individuals make sense of major life experiences, with emphasis on the interpretative processes that connect personal meaning to broader contexts. Compared with thematic analysis, which is primarily descriptive, IPA supports a more idiographic and interpretative reading of participants’ accounts, and unlike grounded theory, IPA does not aim to generate formal theory, but rather to illuminate how participants experience and attribute meaning to phenomena. Transcripts were read and reread, with emergent codes developed inductively and clustered into superordinate themes, and then subordinate themes relating to symptoms and function. Coding and theme development continued iteratively until thematic saturation was reached - the point at which no further codes or themes emerged. To ensure analytic rigour, thematic findings were reviewed through regular team meetings involving qualitative and surgical perspectives. This triangulation ensured interpretations reflected the participants’ narratives rather than the biases or assumptions of a single researcher. Supplementary File 3 is a visual summary of the interview schedule and process.

## Results

Fifteen participants took part in this study (8 men and 7 women) with an age range of 36–85 years (median = 65 years; interquartile range = 30 years (45–75)). Table 1 provides an overview of participant characteristics, and Supplementary File 4 presents individual biographies to offer further context to the narratives explored below.

**Fig. 1:**
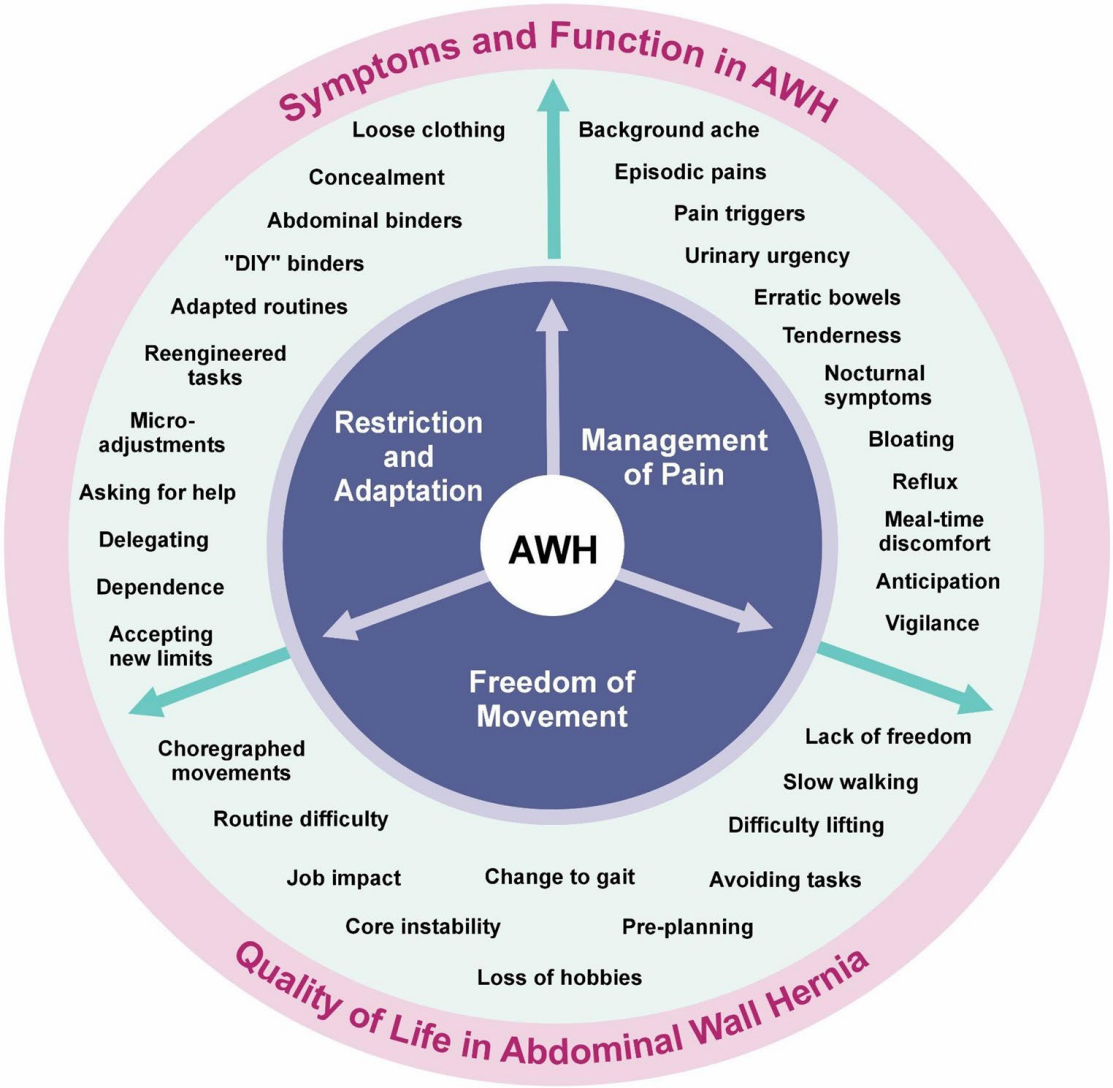
Thematic map illustrating superordinate and subordinate themes concerning the impact of AWH on “symptoms and function”

Analysis of the interviews identified a superordinate theme of “Symptoms and Function”, capturing the ways in which AWH shaped participants’ bodily experiences and day-to-day functioning. Within this superordinate theme, three subordinate themes were identified: *management of pain*, *freedom of movement*, and *restriction and adaptation*. These themes were frequently overlapping and interrelated, reflecting the complex and evolving ways in which participants navigated the physical and psychological demands imposed by their hernia. Figure 1 illustrates the structure and relationship of these thematic domains.

### Subordinate theme one: management of pain

Pain was a central symptom through which participants experienced their hernia. Accounts clustered into two broad patterns, of background pain and episodic exacerbations, often coexisting in the same individual. Participants also described associated visceral and bowel related sensations that were intimately associated with pain, often occurring at the same time or with similar debilitating consequences. Pain, and its associations, led to the following anticipatory vigilance and adaptations in behaviour:

a A persistent, background ache: Many participants lived with a continuous, low-grade discomfort that intensified with activity or as the day progressed. Norman (78) described his hernia as “*just a bit of a nagging pain at times… a dull ache*,* particularly towards night*”, whilst noting how even casual bending brought the sensation into sharp awareness. For Agnes (65) the ache was “*almost like a pulsing*,* constant pain*” that accompanied routine tasks such as gardening or Tai Chi and made her wonder whether she would have to abandon those hobbies altogether. George (45) likewise reported that the ache “*is worse on a night when I’ve had a big meal for my tea*” leaving him with little appetite and a tender abdominal wall around his stoma site.
b Episodic, sharp exacerbations: Superimposed on baseline discomfort were sudden spikes of severe pain. These exacerbations appeared mechanically provoked, often by bending, twisting, lifting, or simply shifting bodily position. Ophelia (44) recalled an incident in which the hernia became trapped after crawling with her children: “*I was in agony… really sharp pain*,* couldn’t stand up properly. It took my breath away*”. These events were not daily but left lasting impressions. Eric (78) described “*I can go for several days with no pain*,* just the lump*,* but then occasionally*,* I don’t know what it is*,* I can’t relate it to food or diet*,* but something seems to set it off and it becomes tender and quite painful*,* and I just have to sort of sit it out.*”. The unpredictability of pain left participants fearful of bowel entrapment each time the pain struck. Nocturnal episodes were described as particularly alarming: Agnes recounted “*waking up in the night literally sweating with the pain… it definitely felt as though something was about to split*”. Uniting these accounts was not only the intense pain, but its inconsistency and suddenness. Such pain contrasted to the slow burning ache that many otherwise managed. When combined, such intensity and prolonged aches produced a persistent anticipatory fear, and participants felt unable to relax, uncertain of when the next spasm would arrive or whether it would signal something more dangerous.
c Visceral and bowel related sensations: Alongside musculoskeletal discomfort, many participants described disturbances in gastrointestinal and urinary function that they attributed to the mass effect or mechanical distortion caused by their hernia. Though visceral symptoms are often considered separately from musculoskeletal pain, participants described them as cooccurring and often indistinguishable from pain. These sensations often amplified their experience of pain or discomfort, and contributed to a pervasive uncertainty about internal bodily signals. For some, urinary urgency and frequency emerged as a new, distressing burden. Agnes associated her nighttime pain with pressure on pelvic organs, describing a new need “*to pee three*,* four times a night*” and erratic, loose bowel motions. Frank (75) similarly reported hourly daytime frequency and nocturia, alongside chronically loose stools that he felt worsened as the hernia enlarged. Gastrointestinal symptoms were equally disruptive. Participants described bloating, acid reflux, loose stools, and even vomiting, often closely linked with activity or eating. George linked mealtime pain to upper abdominal distension and acid reflux, requiring regular proton pump inhibitors, while David (61) recalled bouts of vomiting the day after lifting his toddler granddaughter, which he “*could only assume… was the hernia*”. Several participants found these symptoms unpredictable, unsettling, and ambiguous. Eric (78) remarked on his bowel habit: “*The bowel is really regular… except it’s always loose*” adding that this seemed to have worsened with the growth of his hernia. Participants also highlighted embarrassment associated with the visceral manifestations of hernia. Eric noted, “*There’s nothing between the intestines and the outside world other than a bit of skin really*,* so all the plumbing noises are clearly audible… it can be quite embarrassing at times*.” The psychological impact of audible digestive sounds, leakage, and the need for pads or spare clothing led many to adjust their routines or avoid public spaces entirely.
d Triggers and anticipatory vigilance: Participants quickly learned the movements or situations likely to provoke pain: lifting, twisting, prolonged standing, or even turning in bed. As a result, they approached ordinary tasks with caution, calculating whether the benefit outweighed the risk of a flare. Norman “*was just careful… if lifting and moving anything heavy*” while Lisa (39) found that moving out of a forward bend position invariably set off discomfort, forcing her to adapt the way she tied children’s shoelaces or demonstrated Physical Education (PE) activities. The omnipresent possibility of pain therefore became a lens through which daily life was planned. Strategies participants devised to mitigate or work around this pain, such as bespoke binders or modified movement patterns, are explored in Subordinate Theme 3: Restriction and Adaptation.

Collectively, these narratives illustrate pain as both a chronic backdrop and an episodic threat, intertwined with visceral dysfunction and amplified by fear of catastrophic complications. The result was a heightened bodily vigilance that shaped every decision from meal size to bedtime posture.

### Subordinate theme two: freedom of movement

Loss of physical spontaneity was a pervasive consequence of AWH. Participants described an ever-present awareness of the abdominal mass. This awareness translated into guarded, often reluctant movement and gradually refraining from once valued activities. Describing difficulties with everyday tasks, participants learned to live with their hernia within their work and personal lives. Movement decisions were often determined by anticipation of pain.

a Routine movements were consciously choreographed: Participants described a modification in how they approached simple physical actions. Actions such as dressing, tying shoelaces or exiting a car became multistep manoeuvres, choreographed with care to avoid pain. Lisa (39) explained that “*the bending forward was never a problem; it was coming out of that bend… I had to completely adapt how I did that*”. Betty (63) likened getting down to the bottom of the fridge to “*walking like Mr Blobby… the amount of swearing I do as a result*”, while Eric (78) said that if he dropped something in town he had to “*go down onto one knee to pick it up*” because “*bending is murder*” when strapped into his support brace. Even basic self-care, such as putting on socks or stepping out of the bath, required deliberate pacing and altered body mechanics. These choreographed adaptations reflect heightened bodily consciousness.
b Progressive surrendering of physical tasks: Individuals who once relished strenuous work or sport spoke of retreating and abstaining from them. George (45), working in the trade industry and keen log sawer, confessed, “*I just can’t do anything anymore… I daren’t lift anything*”. Norman (78) acknowledged a progressive change in his experience with hobbies that required physical attention, describing “*we used to play a couple of matches a night well*,* yeah*,* by the time we finished you could feel it there*.”. Betty (63) still walked but only “*with a binder… without it I have to be very careful not to turn a corner too sharply or you end up in a heap on the floor*”. Winter skiing, mountain biking and long haul travel disappeared altogether for some; Eric had “*given that up*” citing twisting forces.
c Disruption to movement impacting both leisure and work: The hernia’s physical limits spilled into roles participants valued. Lisa, a physical education and yoga teacher, stopped trampolining demonstrations because she could not “*get into a safe position… to make sure [students] were safe*”. Eric’s outdoor pursuits career shrank to occasional field archery as he worried about “*nothing between me and being disembowelled*” in a fall. Participants spoke of guilt when colleagues shouldered their duties, often reliant on physical movement, that once anchored their sense of self.
d Vigilance and anticipation with respect to movement: Fear of protrusion, pain or incontinence demanded meticulous pre-planning. Ian spent “*ten minutes… poking [the hernia contents] back in again*” after walks and therefore avoided routes without nearby seating. Agnes carried a mobile and no longer travelled or embarked on hobby activities alone, admitting that instead of watching birds she now calculated “*how far are we from help if I need it*”. Frank (75) packed spare pads and a second brace in his rucksack before striding the city walls because brisk walking could trigger urinary leaks or brace-induced rashes.

Together, these narratives portray freedom of movement as a continuum of forfeited spontaneity. Everyday gestures slowed by conscious sequencing, vigorous pursuits set aside, identities redrawn, and excursions were mapped around contingency plans. The emotional toll and creative adaptations that flowed from this vigilance are explored in Subordinate Theme 3: Restriction and Adaptation.

### Subordinate theme three: restriction and adaptation

Participants rarely accepted restriction passively; instead, they engaged in an ongoing, inventive process of adjustment that touched clothing, household routines, work roles, and interpersonal dynamics. Four interconnected patterns illustrate how they reshaped life around the hernia.

a Clothing, abdominal binders and custom belts: Several participants bypassed standard hospital belts and devised their own solutions. Standard issue hospital binders were often found to be ill-fitting, inflexible, or impractical for daily use. Betty (63) “*started designing my own… cotton jersey … model two*,* which is double bonded jersey*,* and I’ve got three of these*” explaining that commercial binders, whilst “*fantastic for standing and walking*”, dug into her ribs and made driving “*really difficult*”. Eric (78) bought successive internet braces yet still found the latest NHS strap “*massive… quite honestly it restricts your breathing*” and unbearably hot in summer. Many chose loose fitting or “*floaty*” clothes to disguise contour changes: Ophelia (44) wore “*always floaty clothes. Never anything tight*”. Not trivial, these decisions reflected a desire to reclaim control and feel physically safe, even if it meant trading style or comfort, as clothing modifications did not always mask the changes participants saw in their bodies.
b Reengineering of everyday tasks and environments: Participants compartmentalised chores into smaller steps, introduced new tools, or altered body mechanics. Betty described an evening Pilates-style routine “*adapted to try and keep as flexible as possible… it would be easy to sit still and do nothing all day*,* but you’d completely stiffen up*”. She also judged whether she or her husband with sciatica should lift the food processor, noting that “*bending is way more difficult… getting back up again*.” Eric knelt rather than bent when gardening and carried spare braces on long outings so sweat-soaked ones could be swapped midday. Such micro-adjustments permeated cooking, bathing, even using public toilets, with Betty likening removing a Velcro binder in a cubicle to “*trying to get an octopus into a dinner jacket under an anorak*”.
c Renegotiation of personal and social roles around new limits: The multiplication of restrictions contributed to changes in participants’ identity. Lisa (39) catalogued “*an awful lot of adaption in terms of what I was physically able to do”* from abandoning trampolining demonstrations to sitting on the floor to tie children’s shoelaces. David (61) lessened play with his toddler granddaughter, fearing she would remember him as “*too poorly… couldn’t do that with us*”. Frank, a retired soldier, felt compelled to ignore medical advice on weight bearing and carry a coffin at a comrade’s funeral to honour his role despite the hernia’s weight. These adjustments revealed how functional restrictions interrupted their respective identities as athletes, carers, workers, or grandparents.
d Learning to ask for, and accept, help became an accepted norm: Ian (58) spoke of having to “*reprogram your mind that maybe you shouldn’t be lifting that… it’s hard work just asking for help sometimes*”. Participants described relinquishing autonomy and learning to ask for help, and this was most challenging for individuals who had long identified as physically capable or independent. Frank echoed this mental shift: he had to “*learn how to ask for help… to reprogram my mind*”, recognising dependence as a new, if uncomfortable, norm. For some, this meant family members shadowing walks or carrying shopping. David (61) explained that his adult daughter would leave work to accompany him on walks, fearful that he might faint from low energy or pain: “*She’s frightened about if I*,* wherever I go*,* she wants to go to make sure I’m safe*.”

Across these narratives, restriction was not a static deficit but a catalyst for adaptation and ingenuity. Participants stitched new garments, rescripted household chores, recalibrated cherished identities and, often reluctantly, enlisted others’ assistance. Whilst demonstrating much resilience, such adaptations involved burdensome constant vigilance and an implicit acknowledgement that life had irrevocably changed.

## Discussion

Our analysis demonstrates how AWH is experienced as a progressive tightening of patients’ physical radius. Persistent pain, episodic “flares,” and visceral disturbances combine with a loss of spontaneous movement to produce anticipatory vigilance and far-reaching adaptations in work, leisure, dress, and family roles. Over time, patients do not merely *cope*; they rescript daily practices and personal identities around the hernia. The results of this study are visually summarised in a thematic map (Fig. 1).

Pain and visceral discomfort were central to participants’ lived experience of AWH, shaping physical capacity, emotional state and behavioural response. Participants consistently described two distinct pain patterns: a persistent, background ache, and episodic, sharp exacerbations. These latter episodes, marked by acute, sometimes nocturnal pain, were especially distressing due to their unpredictability and perceived threat of rupture or strangulation. Episodic pain was often described as sudden, severe, and mechanically triggered, leaving participants uncertain about its cause. Many attributed these episodes to their hernia “splitting,” or “getting trapped,” reflecting both the physical sensation and the fear of internal damage. Importantly, participants emphasised the unpredictability of these flares: ordinary movements such as bending, coughing, or turning in bed could provoke intense discomfort without warning. This sense of unpredictability fuelled anticipatory vigilance and reinforced a perception of bodily fragility.

In parallel, participants described visceral and urological symptoms that they attributed to the mass effect or spatial distortion caused by the hernia. These findings reflect previous literature that documents a wide range of hernia related symptoms beyond the site of protrusion. In a prospective cohort of patients with clinically confirmed incisional hernia, Ah-Kee et al. found that over 30% of symptomatic individuals reported pain alongside functional limitations, often with multiple complaints simultaneously, such as pain, discomfort, impaired body image, and restriction of activity [14]. Importantly, the presence of irreducible hernias was significantly associated with symptomatology, suggesting that structural severity may correlate with the breadth and intensity of symptoms experienced. Inclusion of these symptoms in surgical assessments may improve holistic preoperative counselling and better characterise the true burden of hernia disease.

Our qualitative findings also lend support to a biopsychosocial understanding [15] of the impact of AWH, wherein physical symptoms initiate a cascade of emotional [16] and social consequences [17]. Participants described persistent pain and reduced mobility not only limiting their physical capacity but also triggering emotional responses such as frustration, anxiety, and a loss of confidence. These emotional burdens, in turn, influenced social behaviour. This led individuals to withdraw from previously valued activities, avoid social situations, or alter their roles within families and communities. The progressive quality of this contraction was striking, with most respondents tracing a personal timeline from “*nagging twinge*” to forfeiting entire sports, careers, or grandparenting rituals, indicating that symptoms and restrictions are dynamic, not static. This process of physical restriction leading to psychosocial adaptation aligns closely with other domains explored in our broader study [7]. For example, themes of body image disturbance and sexual and social relationships reflect similar trajectories; from an initial physical disruption (e.g. hernia visibility or discomfort) to changes in emotional state and interpersonal engagement [9].

Finally, our qualitative findings revealed that pain can trigger a cascading cycle of avoidance and vigilance. Described experiences closely align with the Fear Avoidance Model (FAM), which posits that when pain is perceived as threatening, it elicits hypervigilance and withdrawal from activity. Several participants articulated classical FAM elements, demonstrating progressive disability and disuse [18, 19]: catastrophic anticipation (“*something feels about to split*” - Agnes), activity avoidance (“*I daren’t lift anything*” - George), and perpetuation of pain through de-conditioning. In this model, pain is not merely a nociceptive event but a learned threat response [20], shaped by meaning and context. Because restriction evolved through intertwined physical and cognitive pathways, effective interventions will likely be multimodal. Integrating principles from FAM-informed graded exposure (for example, graded return to twisting, lifting and even coughing) could reduce catastrophic predictions while improving strength. A hernia-specific physiotherapy or structured exercise protocol might progress from seated abdominal bracing to controlled coughing and finally to floor to waist lifting, with each step paired with cognitive re-labelling (“*muscle activation*,* not tearing*”). With respect to surgical intervention, data from a recent prospective cohort study, from Palermo, suggests that postoperative improvements extend beyond symptom relief to include improved quality of life and restored neuromuscular coordination, measured through centre of gravity (barycenter) variation assessment, the Quebec back pain disability scale, and Activities Assessment Scale (AAS) [21].

Binders were described as supportive and often essential for ambulation. For many participants, wearing a binder was not optional. However, participants reported that standard-issues were sometimes hot, rigid, or incompatible with sitting. The do-it-yourself binder culture we observed mirrors similar practices in ostomy communities [22], where patients cut, pad, and Velcro bespoke devices. The act of altering one’s binder was also a quiet assertion of agency. Phenomenologists, such as Leder, speak of the “*dys-appearance*” of the body (distinct from bodily dis-appearance) in which the normally invisible body becomes highly visible when ‘ill’ or ‘bad’ [23]. The herniated abdomen, both felt and seen, disturbed participants’ sense of normalcy. Binders also drew attention to this lost sense of normalcy, and “*dys-appearance*”.

**Fig. 2:**
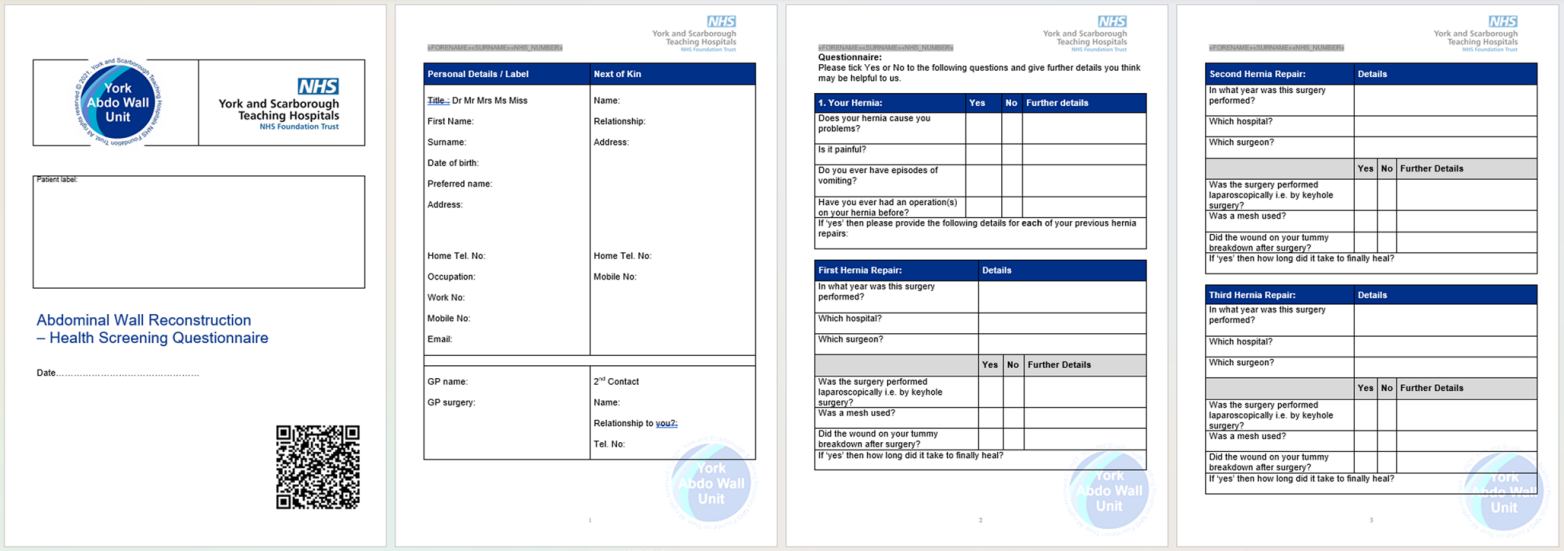
York abdominal wall unit’s “health questionnaire”

Translating these insights into care begins with the consultation. Surgeons can move beyond “*How much does it hurt?*” to ask: “*Which activities have you stopped? How long do you spend preparing for a simple outing? What creative fixes have you invented?”.* These questions legitimise the unseen burden and create opportunity for multidisciplinary solutions. Such multidisciplinary solutions may involve physiotherapy (graded exposure), occupational therapy (ergonomic aids), psychology (fear management), and peer groups (sharing binder “hacks” and clothing tips). To support this shift in consultation dynamics, the York Abdominal Wall Unit has implemented a preconsultation “Health Questionnaire”. Completed by patients at home, this Questionnaire captures granular details of functional limitation, emotional wellbeing, and social impact of their hernia. This approach allows surgeons to allocate more of the consultation to shared decision making, rather than “routine” history-taking. Evidence supports that pre-visit questionnaires can improve data collection and communication between patients and health professionals [24–26]. Figure 2 illustrates selected pages from this questionnaire, and the full Questionnaire can be seen in Supplementary File 5.

### Study limitations

This study employed a phenomenological approach, prioritising rich, first-person narratives to explore how individuals experience and interpret the symptoms and restrictions imposed by AWH. This method relies on participants’ ability to reflect upon, remember, and articulate their experiences. As such, it may miss forms of knowledge that are enacted through the body but go unspoken (for example, subtle compensatory movements or unconscious micro-adjustments). These physical strategies are often habitual rather than narrated and may therefore remain invisible in interview-based data collection. To complement and extend the insights provided by phenomenology, naturalistic observation could offer a valuable methodological addition in future work [27, 28].

The cross-sectional design of this study provides a snapshot in time. While participants often narrated a retrospective trajectory of decline and adaptation, longitudinal research could better trace how symptoms, strategies, and identities evolve over time, or in relation to surgical repair. Tracking these trajectories pre- and post-operatively could help distinguish which adaptations are reversible.

Given the heterogeneity of abdominal wall hernias, we acknowledge that complete saturation across all hernia subtypes cannot be assumed. Our sample predominantly included patients with larger or more complex hernias, reflecting the tertiary referral context. As such, findings may be less transferable to patients with smaller or minimally symptomatic hernias, in whom patterns of restriction and adaptation may differ.

Our participants were drawn from a single, highly-specialised tertiary abdominal wall unit in the UK. Efforts were made, through purposive sampling, to ensure demographic diversity across age, gender, and VWHG grade. However, the views of individuals who cannot access specialist care, or are managed entirely in primary care may differ substantially and warrant further exploration. All interviews were conducted by a single researcher with a clinical background and prior familiarity with abdominal wall hernia care. While this shared knowledge may have facilitated rapport, it may also have introduced implicit biases. Participants may have shaped their narratives based on assumptions about what the interviewer already knew, or what they felt was relevant or appropriate to share in a medical context. To mitigate this, the interviewer engaged in ongoing reflexivity throughout the study.

The role of the interviewer (OS) also warrants consideration. OS is a cispresenting, Caucasian, young, female doctor with surgical and research training, as well as operative experience in CAWH. She had no prior relationship with them before the interviews. Nonetheless, OS engaged in training on cognitive interviewing prior to interviews, and active reflexivity throughout the study, recognising that her demographic and professional background may have shaped how participants related to her, and potentially influenced how the data were interpreted. This reflexive stance was supported by regular analytic discussions with the broader research team, including those outside of surgical practice, to reduce bias and support balanced interpretation.

## Conclusion

This study provides an in-depth phenomenological account of how AWH disrupts bodily function, identity, and daily life. Participants described a variety of physical manifestations of their hernia invading all aspects of daily life. Episodic pain and visceral sensations added layers of unpredictability and distress, contributing to hypervigilance and avoidance. Over time, participants describe choreographing routine activities, avoiding hobbies, and adapting to a new normality. These narratives expose the limitations of traditional, reductionist approaches that assess hernia severity based solely on size or reducibility. Instead, they aid the call for a biopsychosocial model in which pain, function, emotional impact, and adaptive behaviours are viewed as interconnected. Interventions must match the complexity of this lived experience by including multimodal approaches, integrating physiotherapy, psychology, occupational therapy, and patient-led peer support. Such an approach would begin with surgeons asking patients how their hernia affects their daily life. Ultimately, AWH is experienced as a diffuse, *cacophonous* disruption, and a care model that acknowledges this complexity is vital, and will restore confidence, capability, and selfhood in those living with hernia.

## Supplementary Information

Below is the link to the electronic supplementary material.

Supplementary Material 1 (DOCX 28.9 KB)

Supplementary Material 2 (DOCX 26.5 KB)

Supplementary Material 3 (DOCX 622 KB)

Supplementary Material 4 (DOCX 29.2 KB)

Supplementary Material 5 (DOCX 2.68 MB)
