# Supplementary material for: “I just can’t do that anymore”: a qualitative exploration of symptoms and function in patients living with abdominal wall hernia (AWH)
Source: Hernia. 2025 Oct 14;29(1):296. doi: 10.1007/s10029-025-03489-3 (PMC12521324; doi:10.1007/s10029-025-03489-3)
Supplement: Supplementary file 1 — Supplementary Material 1 (DOCX 28.9 KB) [file 10029_2025_3489_MOESM1_ESM.docx]

**Supplementary File 1**: Study participant demographics^1^

| Participant name | Sex | Age | VHWG Grade | hernia Height x width (cm) | NHS/  private | Smoker | Diabetic | Wound infection | Stoma | Cancer | Fistula | bmi | Socioeconomic class | employed | Post op/pre op | Telephone interview |
| --- | --- | --- | --- | --- | --- | --- | --- | --- | --- | --- | --- | --- | --- | --- | --- | --- |
| Agnes | F | 65 | 2 | 17x17 | NHS | Ex-smoker | No | No | No | Yes | No | 29.6 | Middle | Yes | Pre-op | No |
| Betty | F | 63 | 1 | 30x20 | Private | Never | No | No | No | No | No | 26.1 | Upper | Retired | Pre-op | No |
| Charlotte | F | 68 | 4 | 30x20 and  60x12 | NHS | Ex-smoker | Yes | Yes | No | No | No | 38.6 | Middle | Retired | Pre-op | No |
| David | M | 61 | 2 | 7x7 and  9x9 | NHS | Never | Yes | No | No | No | No | 31.2 | Lower | Yes | Pre-op | Yes |
| eric | M | 78 | 1 | 12x30 | NHS | Ex-smoker | No | No | No | No | No | 29.9 | Middle | Retired | Pre-op | Yes |
| Frank | M | 75 | 4 | 27x26 | NHS | Ex-smoker | No | Yes | No | Yes | Yes | 25.8 | Middle | Retired | Post-op | Yes |
| George | M | 45 | 4 | 7x8 and  9x13 and  16x20 | NHS | Ex-smoker | No | Yes | Yes | No | No | 30.5 | Lower | Yes | Pre-op | Yes |
| harry | M | 84 | 2 | 23x15 | NHS | Ex-smoker | Yes | No | No | Yes | No | 28.8 | Upper | Yes | Post-op | Yes |
| ian | M | 58 | 4 | 17x21 | NHS | Ex-smoker | No | Yes | No | Yes | No | 30.2 | Middle | Yes | Pre-op | Yes |
| joan | F | 75 | 3 | 22x18 and  19x17 and  Parastomal | NHS | Never | No | No | Yes | Yes | No | 26.3 | Middle | Retired | Pre-op | Yes |
| KEVIN | M | 74 | 3 | 20x15 and 15x15 | NHS | Ex-smoker | No | No | Yes | No | No | 32.4 | Middle | Retired | Post-op | Yes |
| LISA | F | 39 | 1 | 10x12 | NHS | Never | No | No | No | No | No | 29.2 | Middle | Yes | Post-op | Yes |
| MARGE | F | 36 | 1 | 5x20 | NHS | Never | No | No | No | No | No | 20.4 | Middle | Yes | Post-op | Yes |
| NORMAN | M | 77 | 3 | 30x20 | NHS | Ex-smoker | Yes | No | No | Yes | No | 24.1 | Middle | Retired | Post-op | Yes |
| OPHELIA | F | 44 | 1 | 2x3 and 4x5 and 2x3 | NHS | Never | No | No | No | No | No | 30.7 | Middle | Yes | Post-op | Yes |

^1^Names given to participants are pseudonyms, ensuring anonymity
