# Supplementary material for: “I just can’t do that anymore”: a qualitative exploration of symptoms and function in patients living with abdominal wall hernia (AWH)
Source: Hernia. 2025 Oct 14;29(1):296. doi: 10.1007/s10029-025-03489-3 (PMC12521324; doi:10.1007/s10029-025-03489-3)
Supplement: Supplementary file 2 — Supplementary Material 2 (DOCX 26.5 KB) [file 10029_2025_3489_MOESM2_ESM.docx]

**Supplementary file 2:** Interview Topic Guide

**Introduction:**

- Thank you for seeing me today and offering to take part in this study.
- I would like first to outline the study so that you are able to decide whether you wish to proceed further (recap information sheet).
- I have a list of topics that I want to address.
- Feel free to ask questions at any stage during the interview.
- I might make a few notes in case I want to come back to something later.
- A Trust Dictaphone will be used to record our conversation as per the information sheet and consent form. The reason I am doing this is because I will be recording this interview, so I have something to help me remember accurately what we talk about today, the only people who will hear the recording are myself and an independent medical secretary who works for the surgical team and will transcribe the tape, is this ok?
- The recorded information will be anonymous with no names mentioned and it will be secured under lock and key in a designated area in York Hospital. The data will be destroyed after the study.
- If there is anything you find you do not wish to talk about, please let me know.
- I will aim to follow your lead in terms of what we discuss, but if we do stray on to a topic that you are not keen to talk about, tell me straight away and we can discuss something else.
- We can stop the interview whenever you like. If you would like to take a break, or feel upset or unwell, please let me know and we will suspend or stop the interview entirely.
- The interview will last approximately 1-1.5 hours
- Sign consent form × 2 (one for participant and information sheet, one for interviewer)/check signed.

**The Interview:**

The aim of this interview is to discuss your personal experiences of having a complex abdominal wall hernia and how this affects your quality of life. There are no ‘right’ or ‘wrong’ answers, I am interested in how *you view* *your* complex hernia, *your* experiences and how you think this has affected your quality of life. I am now going to start the recording.

Questions:

Firstly, I would like to get to know you a little. Could you tell me a little about yourself?

[aim to put participant at ease]

**Prompts:**

What do you do?

What does a normal day/week look like?

1. Can you tell me a little about your experience living with a complex abdominal wall hernia?

**Prompts:**

Do you experience any difficulties/problems from your hernia?

Can you tell me about those difficulties/problems?

What is bothering you about them?

Could you tell me about the most recent issue that you have had with your hernia?

How did this make you feel?

1. Topics:
   1. **Pain**

What is you experience of pain related to your hernia?

Prompts:

Regarding living with the pain, can you tell me what the most relevant aspect of this is for you in particular?

How does pain affect your daily life?

Does the pain that you experience from your hernia affect your relationships/employments etc. in any way? Can you tell me more about this?

- 1. **Body image/Self perception**

How comfortable are you with what you look life/are you with your physical appearance?

Prompts:

How does the appearance of your hernia affect you?

How does the appearance of your hernia affect your relationship with your partner/friends?

Does this affect any other aspects of your daily life, for example, purchasing clothing?

- 1. **Mental health/happiness**

What about your overall wellbeing? Would you say that your hernia has had any particular positive or negative psychological impact on you?

How does this affect you in your daily life?

- 1. **Employment**

What is your job? What does it consist of?

Prompts:

How does your hernia affect you at work?

Do you think that your hernia has affected your ability to do this job in any way?

- 1. **Physical activities/general activities**

Describe the things you enjoy doing in your daily life.

Prompts:

Do you think that your hernia has affected your ability to do any of these things? If so, in what way.

Can you tell me about any things that you no longer do as a result of your hernia?

What activities do you do for exercises?

- 1. **Relationships and belonging**

Can you tell me about any relationship difficulties have you encountered as a result of your hernia?

Has your hernia affected your relationship with your spouse or partner? What about your friends? What about your family? Can you tell me more about this?

Can you tell me about a time where your hernia has affected a moment with a spouse/family/friend?

Sometimes, people who have complex abdominal wall hernia struggle to have sexual intercourse. Is this something that has been an issue for you?

- 1. **Quality of life**

What does quality of life mean to you?

Do you think your hernia has altered your quality of life?

How has your outlook on life changed as a person living with a complex abdominal wall hernia?

- 1. **Hope**What do you think would improve your experience related to your hernia and your QoL?

[Follow up questions as appropriate. Be led by patient answers.]

Is there anything else you wish to share with me?

Is there anything you further you would like to share about your hernia and how it affects and your quality of life?

**End of interview:**

- I have no more questions today, but I’d like to give you the opportunity to say anything else about your experience with having a complex abdominal wall hernia and how this has affected your quality of life or, concerning anything else we’ve discussed today.
- *Outline what will happen next:* (1) the recording will be typed up and anonymised, then analysed alongside all the other interviews, (2) when the study is complete I will send you a summary of the results unless you would prefer that I didn’t.
- Thank you for taking part in the interview today.
