# Supplementary figures and images for: “I just can’t do that anymore”: a qualitative exploration of symptoms and function in patients living with abdominal wall hernia (AWH)
Source: Hernia. 2025 Oct 14;29(1):296. doi: 10.1007/s10029-025-03489-3 (PMC12521324; doi:10.1007/s10029-025-03489-3)

**Supplementary File 3:** interview schedule


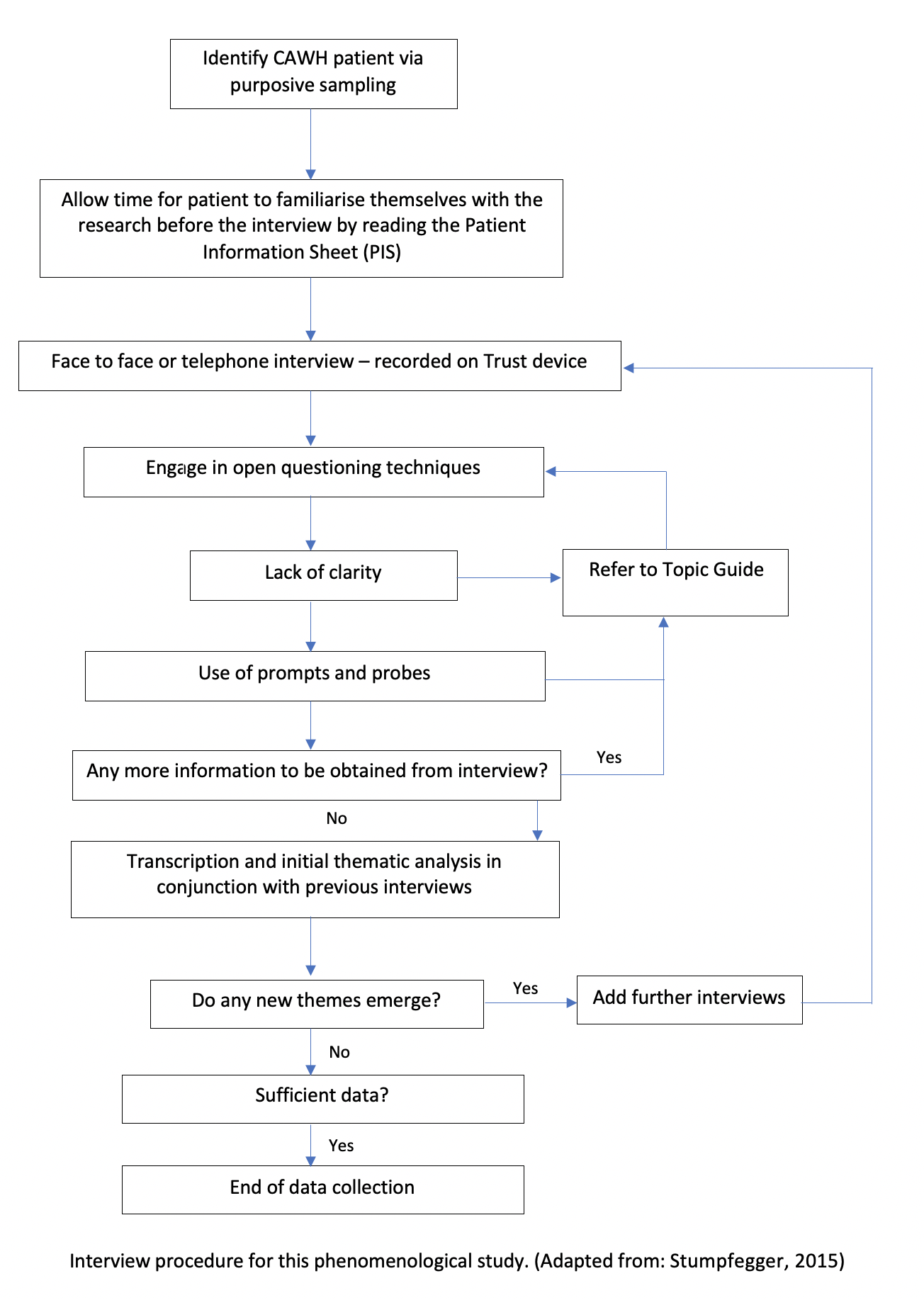

Supplement: Supplementary file 3 — Supplementary Material 3 (DOCX 622 KB) [file 10029_2025_3489_MOESM3_ESM.docx]
