# Supplementary material for: “I just can’t do that anymore”: a qualitative exploration of symptoms and function in patients living with abdominal wall hernia (AWH)
Source: Hernia. 2025 Oct 14;29(1):296. doi: 10.1007/s10029-025-03489-3 (PMC12521324; doi:10.1007/s10029-025-03489-3)

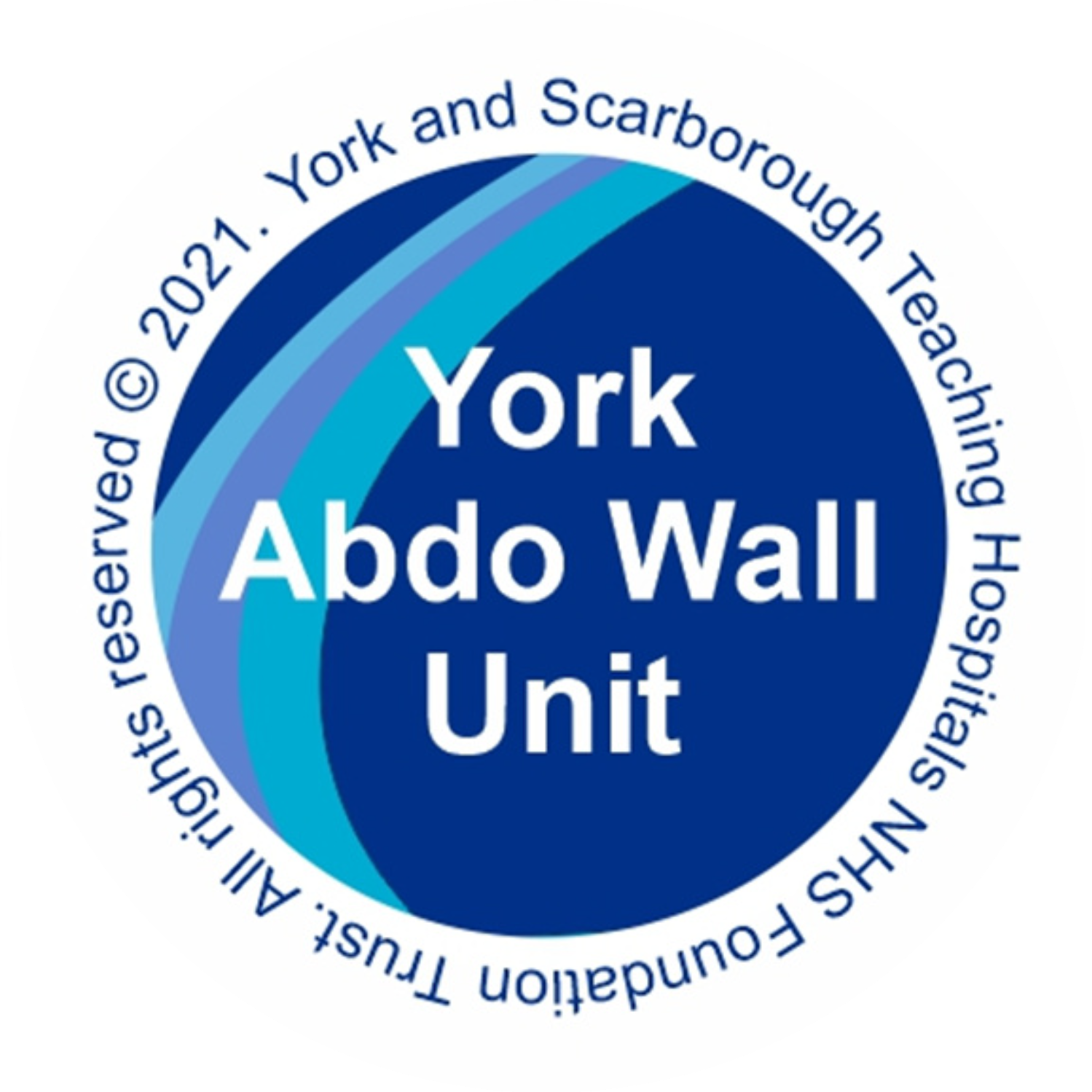


|  | 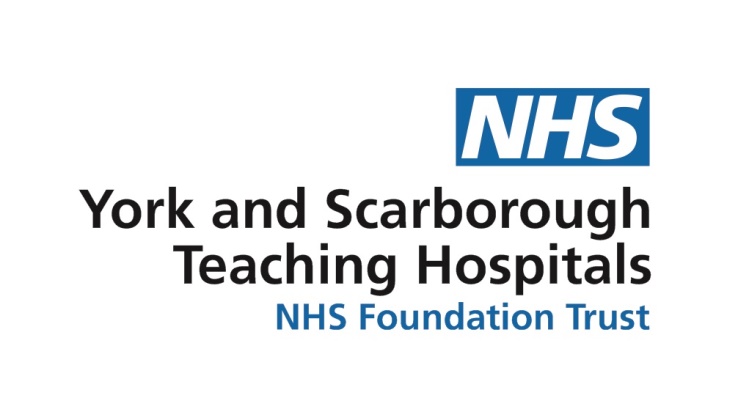 |
| --- | --- |

| Patient label: |
| --- |

Abdominal Wall Reconstruction

– Health Screening Questionnaire


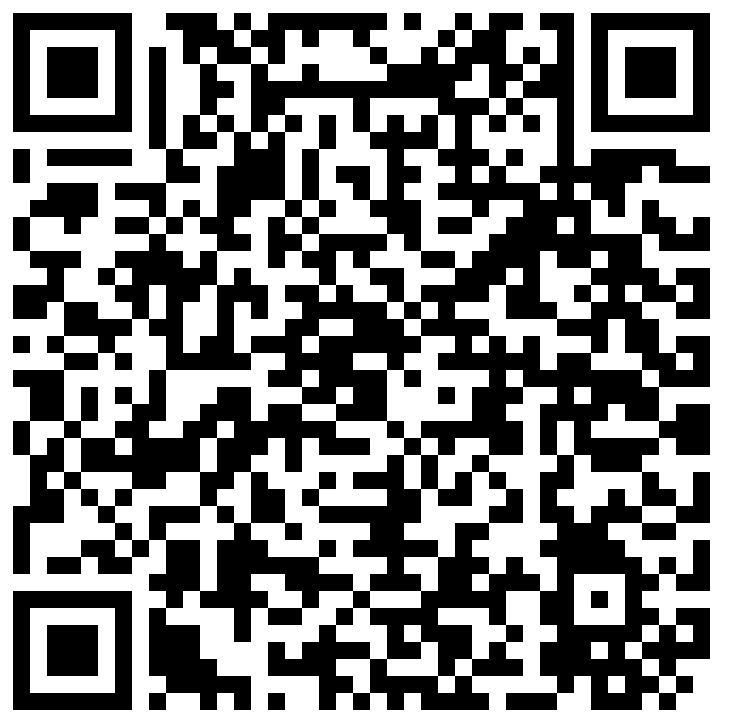
Date…………………………………………

| **Personal Details / Label** | **Next of Kin** |
| --- | --- |
| Title : Dr Mr Mrs Ms Miss  First Name:  Surname:  Date of birth:  Preferred name:  Address:  Home Tel. No:  Occupation:  Work No:  Mobile No:  Email: | Name:  Relationship:  Address:  Home Tel. No:  Mobile No: |
|  | |
| GP name:  GP surgery: | 2^nd^ Contact  Name:  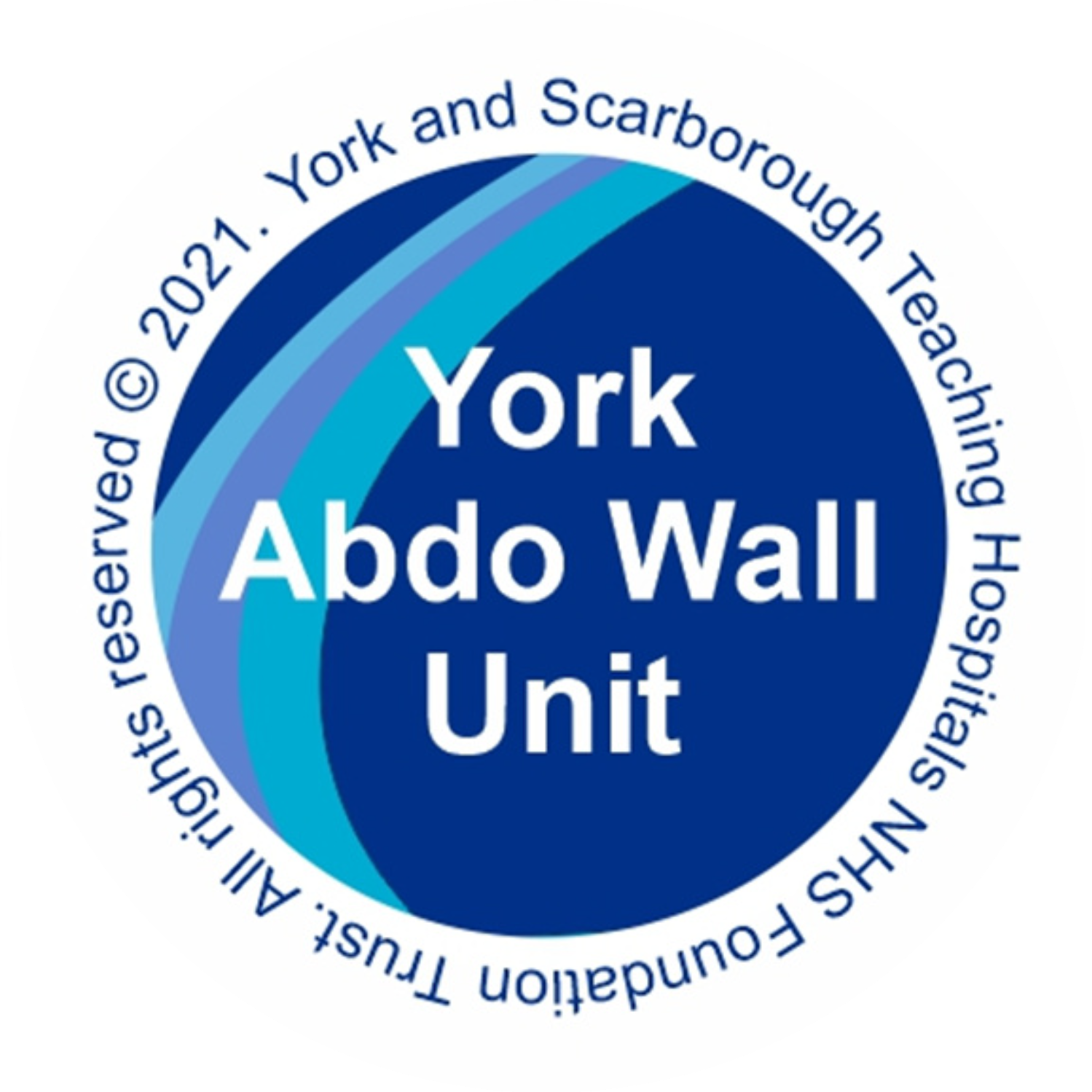Relationship to you?:  Tel. No: |

**Questionnaire:**

Please tick Yes or No to the following questions and give further details you think may be helpful to us.

| **1. Your Hernia:** | **Yes** | **No** | **Further details** |
| --- | --- | --- | --- |
| Does your hernia cause you problems? |  |  |  |
| Is it painful? |  |  |  |
| Do you ever have episodes of vomiting? |  |  |  |
| Have you ever had an operation(s) on your hernia before? |  |  |  |
| If ‘yes’ then please provide the following details for **each** of your previous hernia repairs: | | | |

| **First Hernia Repair:** | **Details** | | |
| --- | --- | --- | --- |
| In what year was this surgery performed? |  | | |
| Which hospital? |  | | |
| Which surgeon? |  | | |
|  | **Yes** | **No** | **Further Details** |
| Was the surgery performed laparoscopically i.e. by keyhole surgery? |  |  |  |
| Was a mesh used? |  |  |  |
| Did the wound on your tummy breakdown after surgery? |  |  | 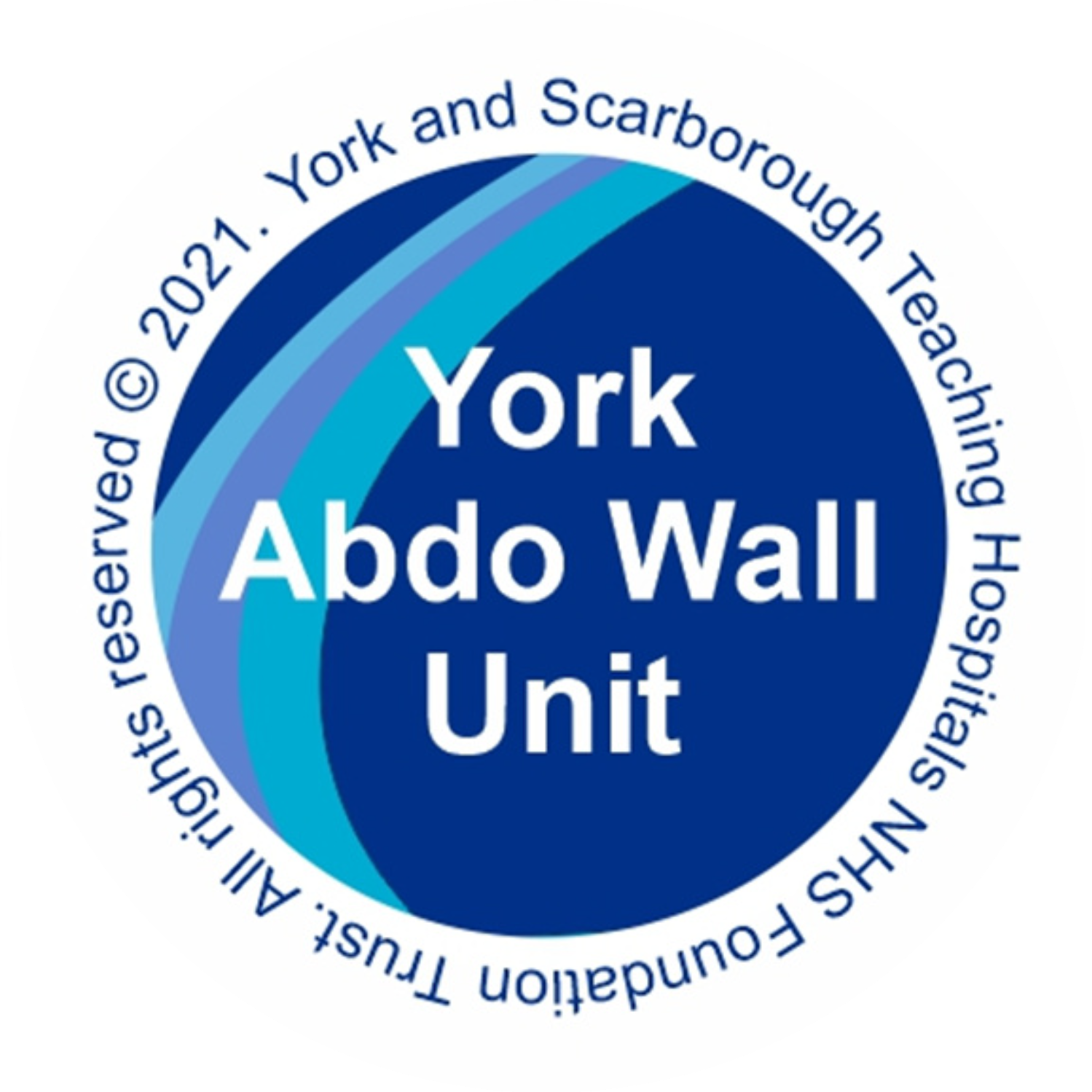 |
| If ‘yes’ then how long did it take to finally heal? | | | |

| **Second Hernia Repair:** | **Details** | | |
| --- | --- | --- | --- |
| In what year was this surgery performed? |  | | |
| Which hospital? |  | | |
| Which surgeon? |  | | |
|  | **Yes** | **No** | **Further Details** |
| Was the surgery performed laparoscopically i.e. by keyhole surgery? |  |  |  |
| Was a mesh used? |  |  |  |
| Did the wound on your tummy breakdown after surgery? |  |  |  |
| If ‘yes’ then how long did it take to finally heal? | | | |

| **Third Hernia Repair:** | **Details** | | |
| --- | --- | --- | --- |
| In what year was this surgery performed? |  | | |
| Which hospital? |  | | |
| Which surgeon? |  | | |
|  | **Yes** | **No** | **Further Details** |
| Was the surgery performed laparoscopically i.e. by keyhole surgery? |  |  |  |
| Was a mesh used? |  |  | 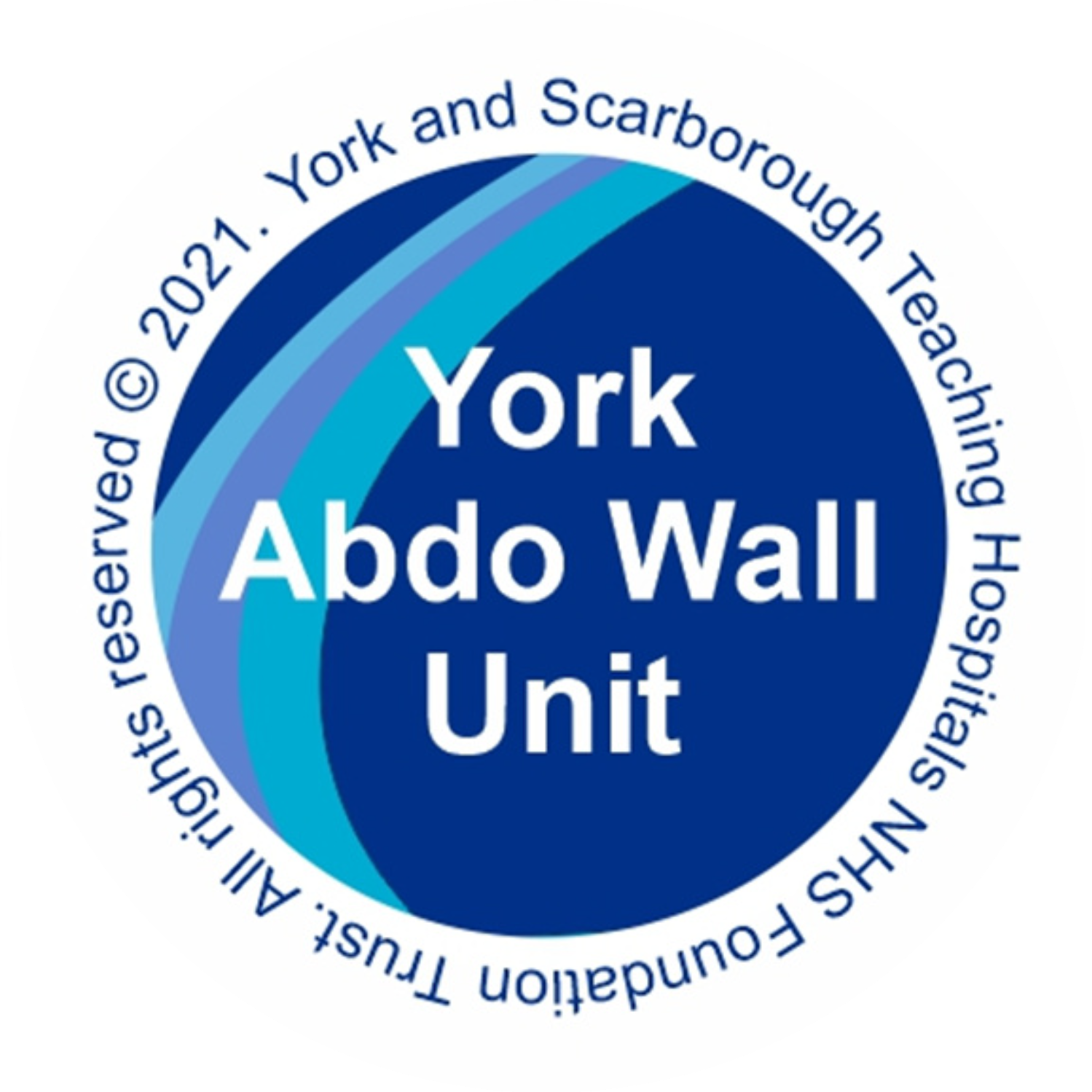 |
| Did the wound on your tummy breakdown after surgery? |  |  |  |
| If ‘yes’ then how long did it take to finally heal? | | | |

| **Fourth Hernia Repair:** | **Details** | | |
| --- | --- | --- | --- |
| In what year was this surgery performed? |  | | |
| Which hospital? |  | | |
| Which surgeon? |  | | |
|  | **Yes** | **No** | **Further Details** |
| Was the surgery performed laparoscopically i.e. by keyhole surgery? |  |  |  |
| Was a mesh used? |  |  |  |
| Did the wound on your tummy breakdown after surgery? |  |  |  |
| If ‘yes’ then how long did it take to finally heal? | | | |


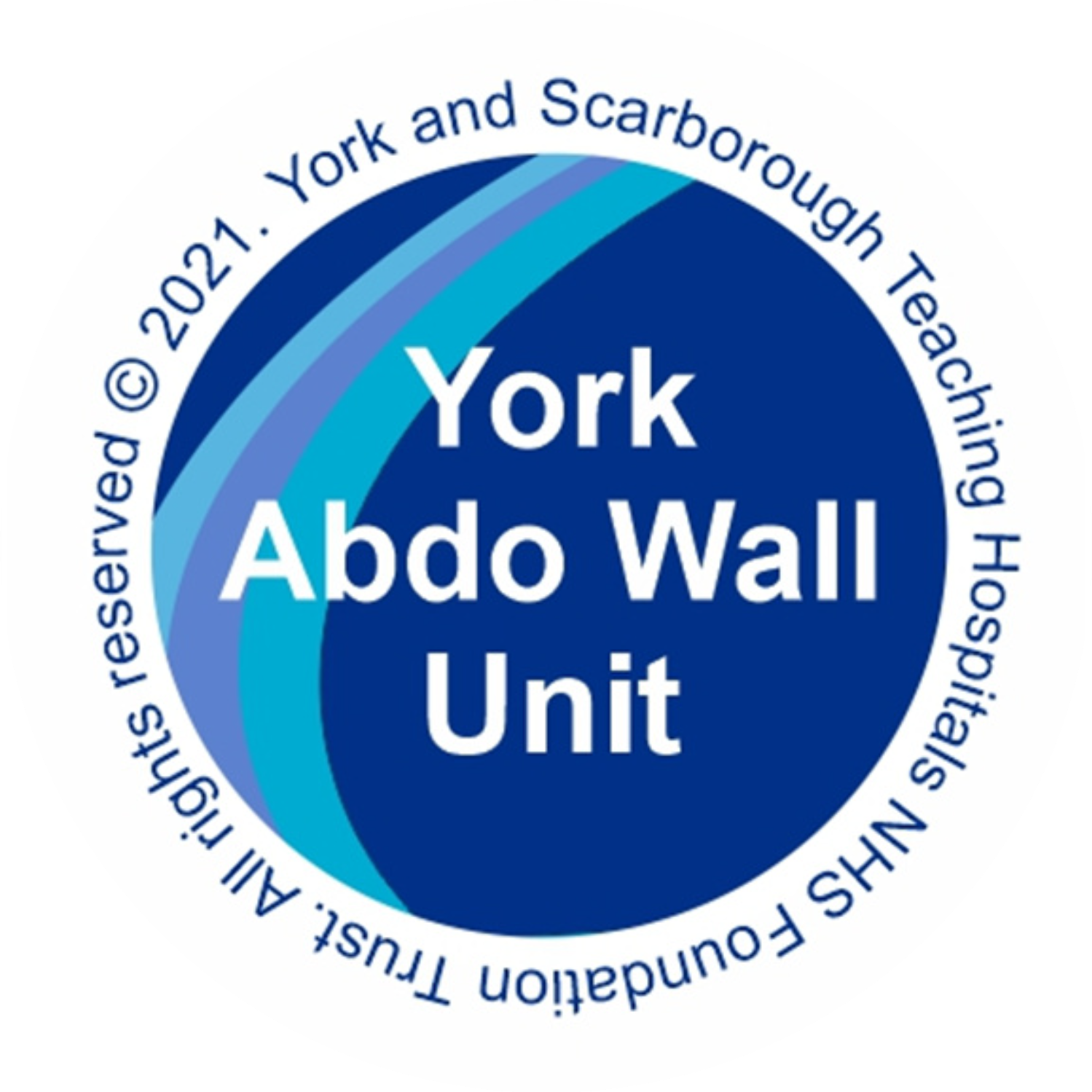


| **3. Previous Operations & Anaesthetics** |  |  |  |  | |
| --- | --- | --- | --- | --- | --- |
| Please give details of any operations that you have had? | | | | | |
| **Operation:** | | | | **Hospital**  **And Surgeon** | **Year** |
|  | | | | 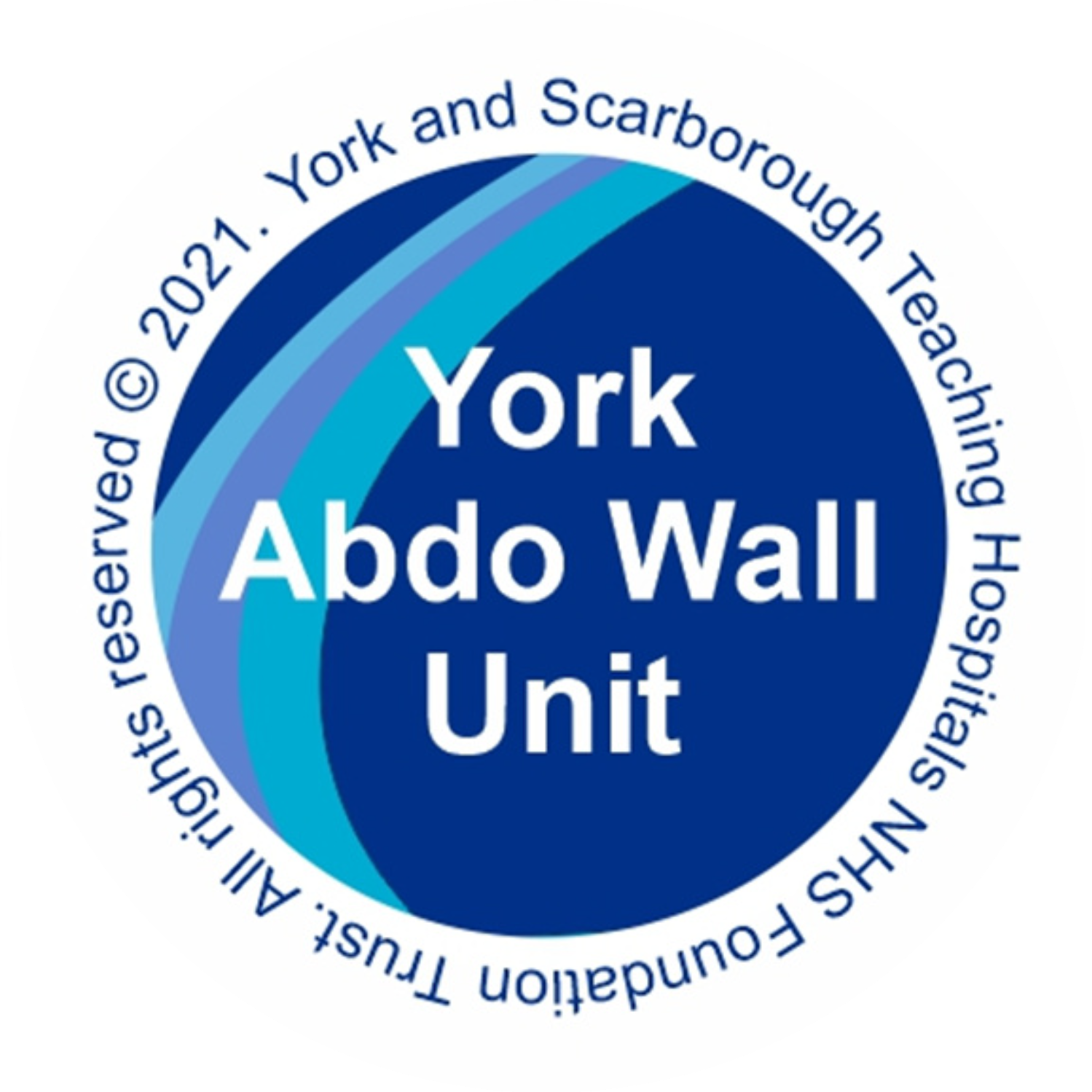 |  |
|  |  | **Yes** | **No** | **Further details** | |
| Have you ever had any problems with any previous anaesthetics? | |  |  | If ‘yes’ please give details | |
| Have any of your relatives had problems with anaesthetics? | |  |  | If ‘yes’ please give details | |

| **4. Body Weight** | **Yes** | **No** | **Further details** |
| --- | --- | --- | --- |
| What is your current weight? |  | | |
| Do you feel that you are overweight? |  |  |  |
| Have you tried to lose weight before? |  |  |  |
| What is the lowest weight you have been as an adult? | | | |
| What is the highest weight you have been as an adult? | | | |
| Is your weight: ☐ going up ☐ staying the same ☐ going down ☐ unsure | | | |

| **5. Activities / Exercise** | **Yes** | **No** | **Further details** |
| --- | --- | --- | --- |
| Are you working at the moment? |  |  |  |
| If ‘yes’ what kind of work do you do? | | | |
| How many times a week are you active for at least 30 minutes?  e.g. walking, swimming, gardening? | | | |
| Do you think that you could walk a mile? |  |  | 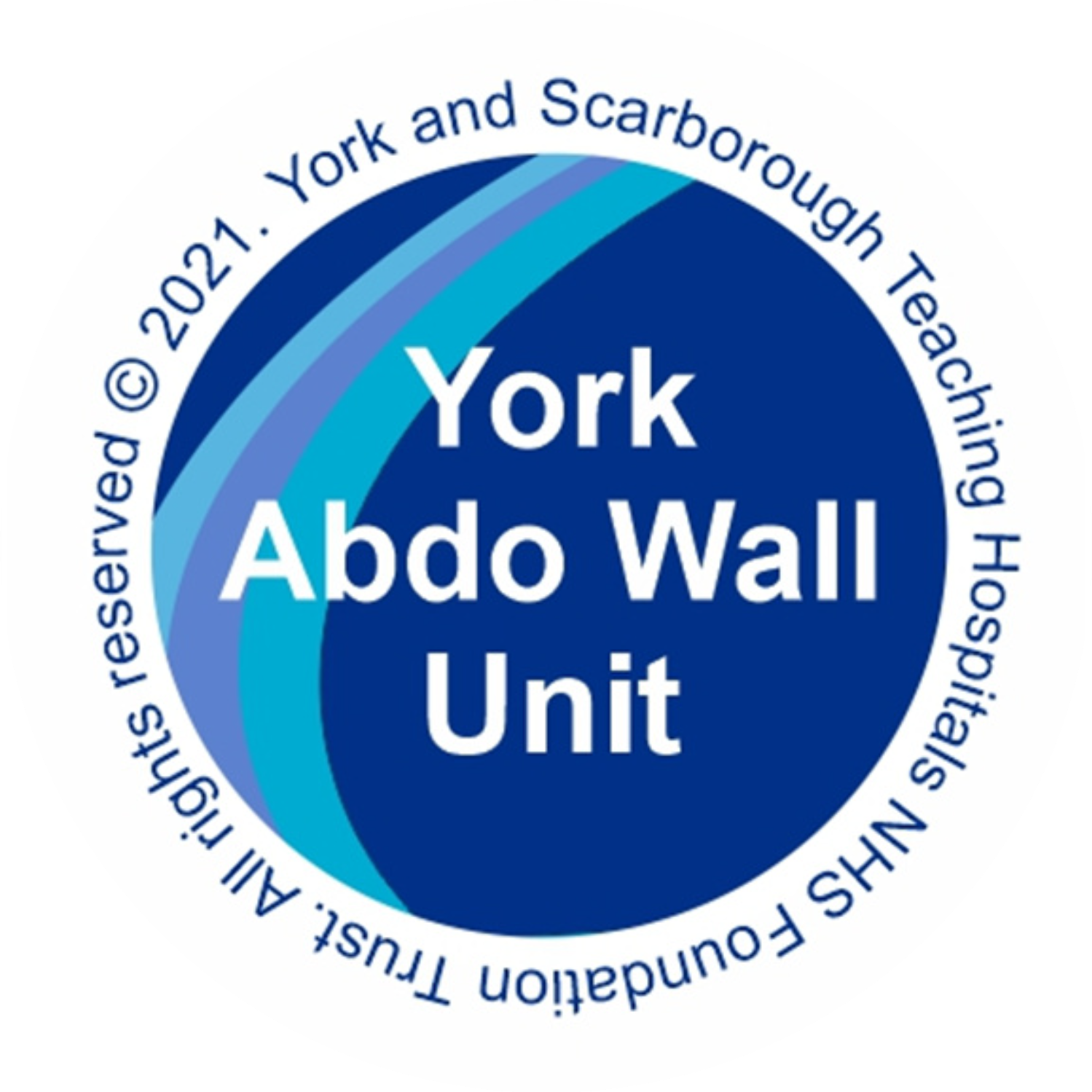 |
| If ‘no’ what stops you from walking e.g. pain (where), breathless, etc. and how far can you walk? | | | |
| Do you exercise regularly? |  |  |  |

| If ‘yes’ how often and describe the exercise. | | | |
| --- | --- | --- | --- |
| Have you thought about exercising as a way to improve your health and fitness? |  |  |  |
| How much time do you spend, during a week, sitting or lying i.e. not active? | | | |
| Do you use a mobility aid (e.g. sticks, walking frame or wheelchair)? |  |  |  |


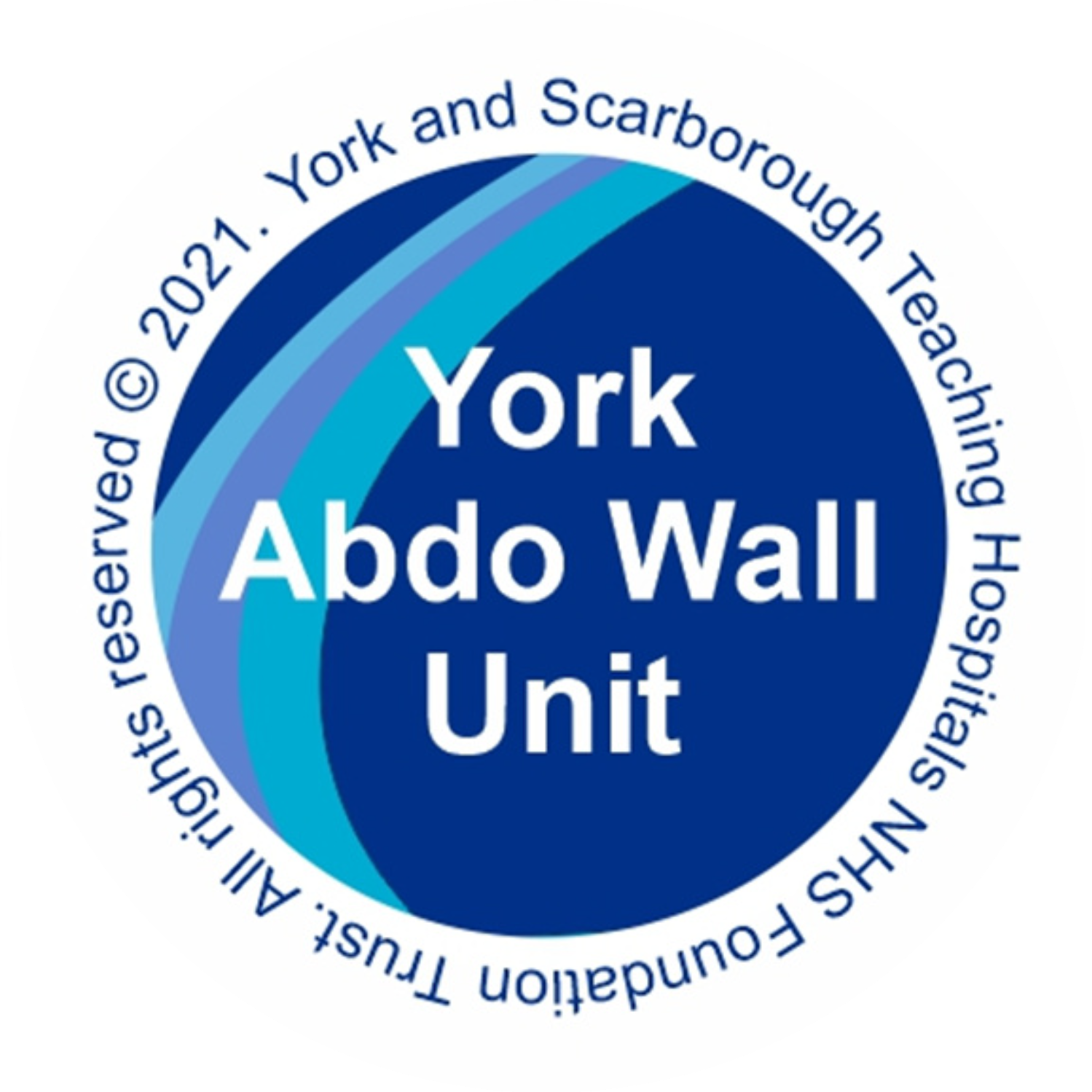


| **6. Diabetes** | **Yes** | **No** | **Further details** |
| --- | --- | --- | --- |
| Do you have diabetes (diabetes mellitus)? |  |  |  |
| If ‘yes’ are you treated with insulin or tablets? |  |  |  |

| **7. Immunity** | **Yes** | **No** | **Further details** |
| --- | --- | --- | --- |
| Are you immunosuppressed? |  |  |  |
| Do you take steroids? |  |  |  |
| Have you ever been diagnosed as having any type of cancer? |  |  |  |
| Was this treated with chemotherapy or radiotherapy or both? | | | |

| **8. Smoking** | **Yes** | **No** | **Further details** |
| --- | --- | --- | --- |
| Do you smoke now? |  |  |  |
| If ‘yes’ – how much do you smoke a day and for how many years? |  |  |  |
| If ‘yes’ would you like to give up? |  |  |  |
| If ‘no’ did you used to smoke? |  |  |  |
| If you used to smoke, when did you give up? | | | |
| How much did you used to smoke? | | | |


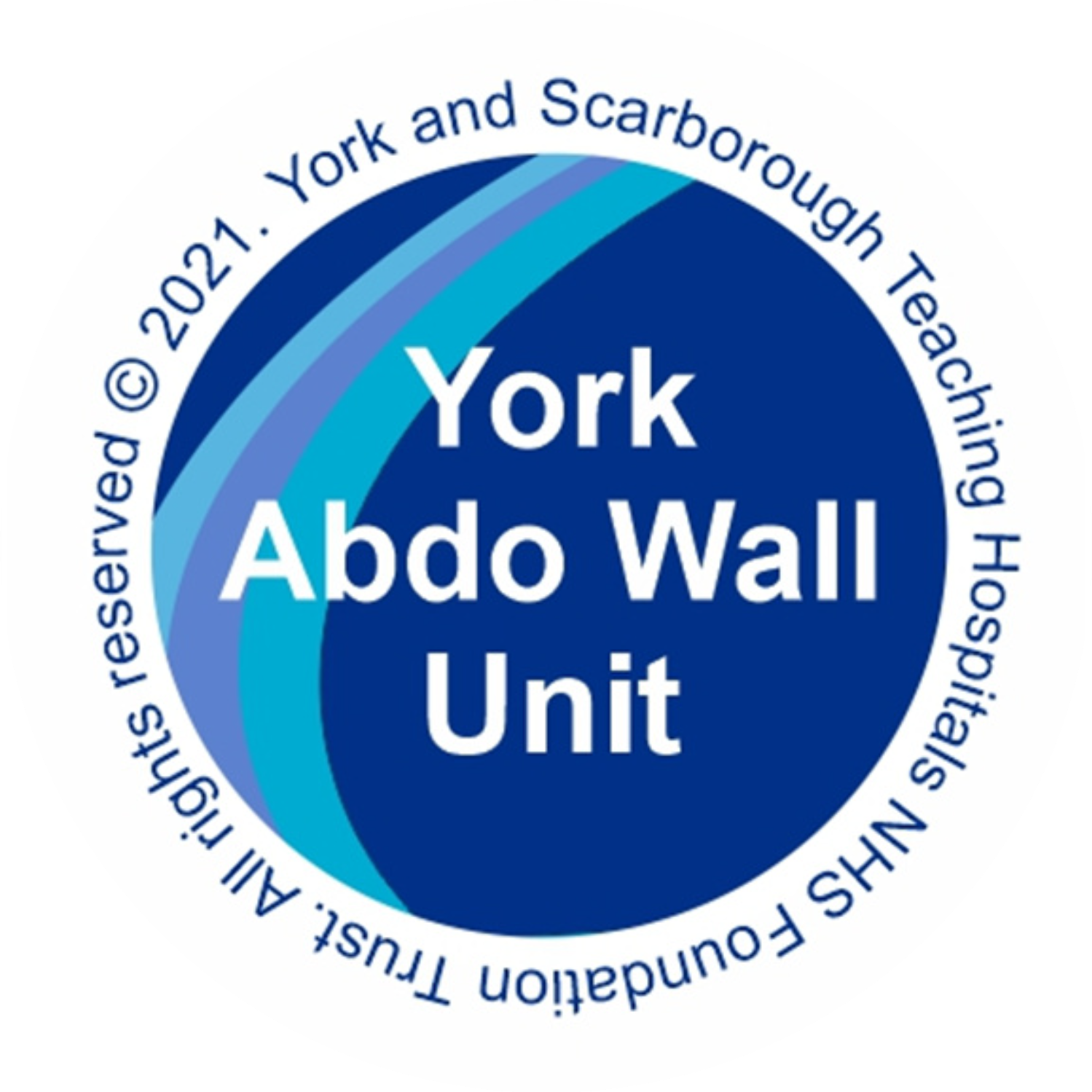


| **9. Infection** | **Yes** | **No** | **Further details** |
| --- | --- | --- | --- |
| Have you had any abdominal wound infections in the past? |  |  |  |
| If ‘yes’ please give details | | | |
| Have you ever suffered a serious infection (e.g. MRSA, clostridium difficile? |  |  |  |
| If ‘yes’ please give details | | | |
| Do you currently have a stoma? |  |  |  |
| Do you currently have a bowel fistula? |  |  |  |
| Do you currently have any open wounds / ulcers / blisters? |  |  |  |

| **10. Breathing Disorders** | **Yes** | **No** | **Further details** |
| --- | --- | --- | --- |
| Do you have asthma, chronic obstructive airways disease (COPD) or any other breathing disorder? |  |  |  |
| How many hospital admissions have you had in the last 12 months? | | | |
| Have you ever been admitted to the intensive care unit because of your breathing? |  |  |  |
| If ‘yes’ please give details e.g. did you need a tracheostomy | | | |
| Do you use inhalers and/or nebulisers at home? |  |  |  |
| Do you use home oxygen? |  |  |  |
| Do you have sleep apnoea? |  |  | 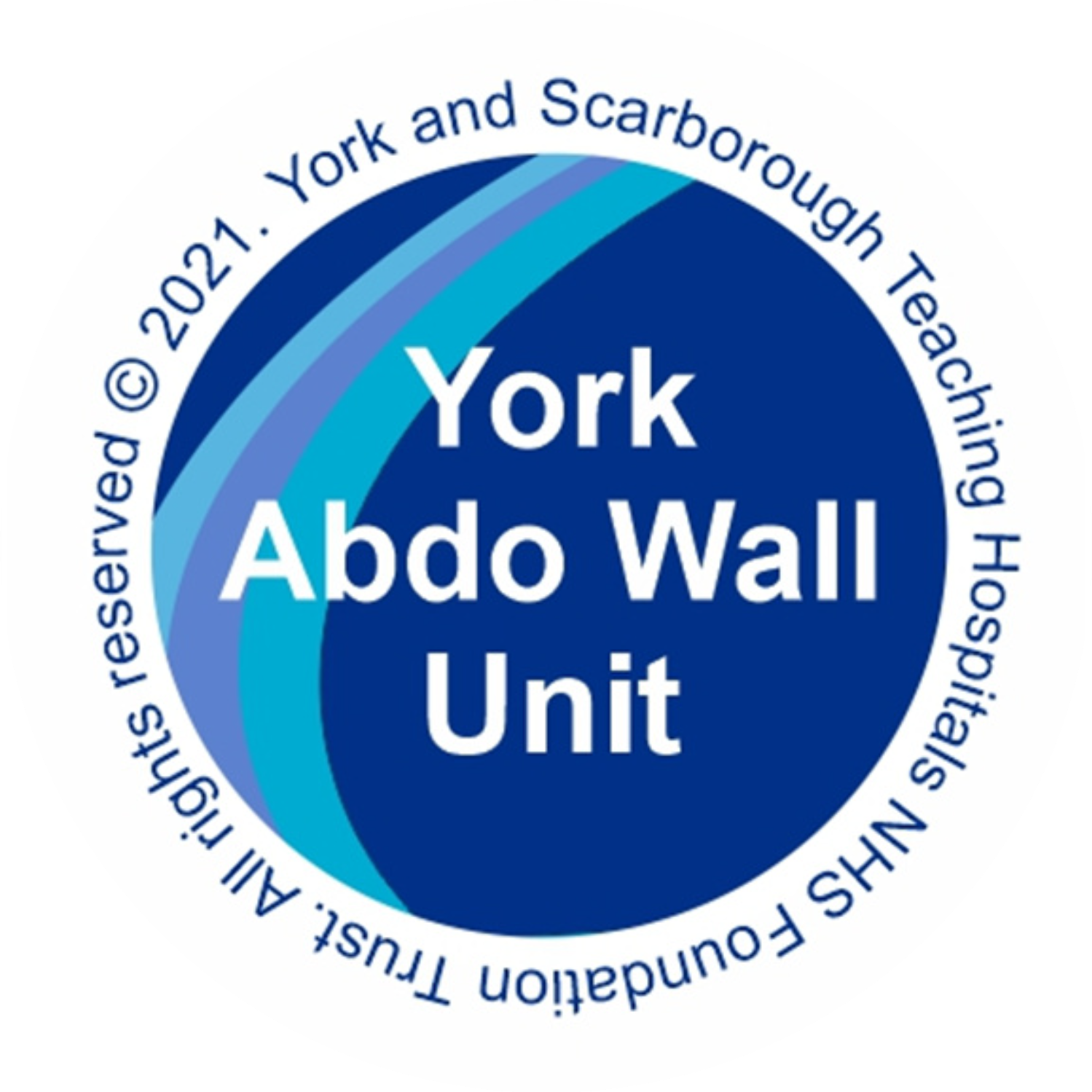 |
| Do you use a CPAP machine at night? |  |  |  |
| If ‘yes’ how long you have used CPAP for, and how many hours a night do you use it for? | | | |

| **11. Heart Disease** | **Yes** | **No** | **Further details** |
| --- | --- | --- | --- |
| Do you get chest pain or become breathless climbing two flights of stairs? |  |  |  |
| Do you suffer with angina? |  |  |  |
| Have you had a heart attack? If ‘yes’ please give year |  |  |  |
| Have you had angioplasty (a balloon to open up a blocked artery) or heart bypass surgery? |  |  |  |
| If “yes” please give details | | | |
| Do you have coronary stents? |  |  |  |
| Are you currently being treated for an irregular heartbeat |  |  |  |
| Have you ever been treated for heart failure? |  |  |  |
| Have you ever been told that you have a heart murmur? |  |  |  |
| Are you being treated for high blood pressure? |  |  |  |
| Do you have a pacemaker or an implanted defibrillator? |  |  |  |

| 12. Hormone, renal, liver & bleeding disorders | | Yes | No | Further details | |
| --- | --- | --- | --- | --- | --- |
| Do you have thyroid disease? | |  |  |  | |
| Have you ever been diagnosed with kidney disease? | |  |  |  | |
| 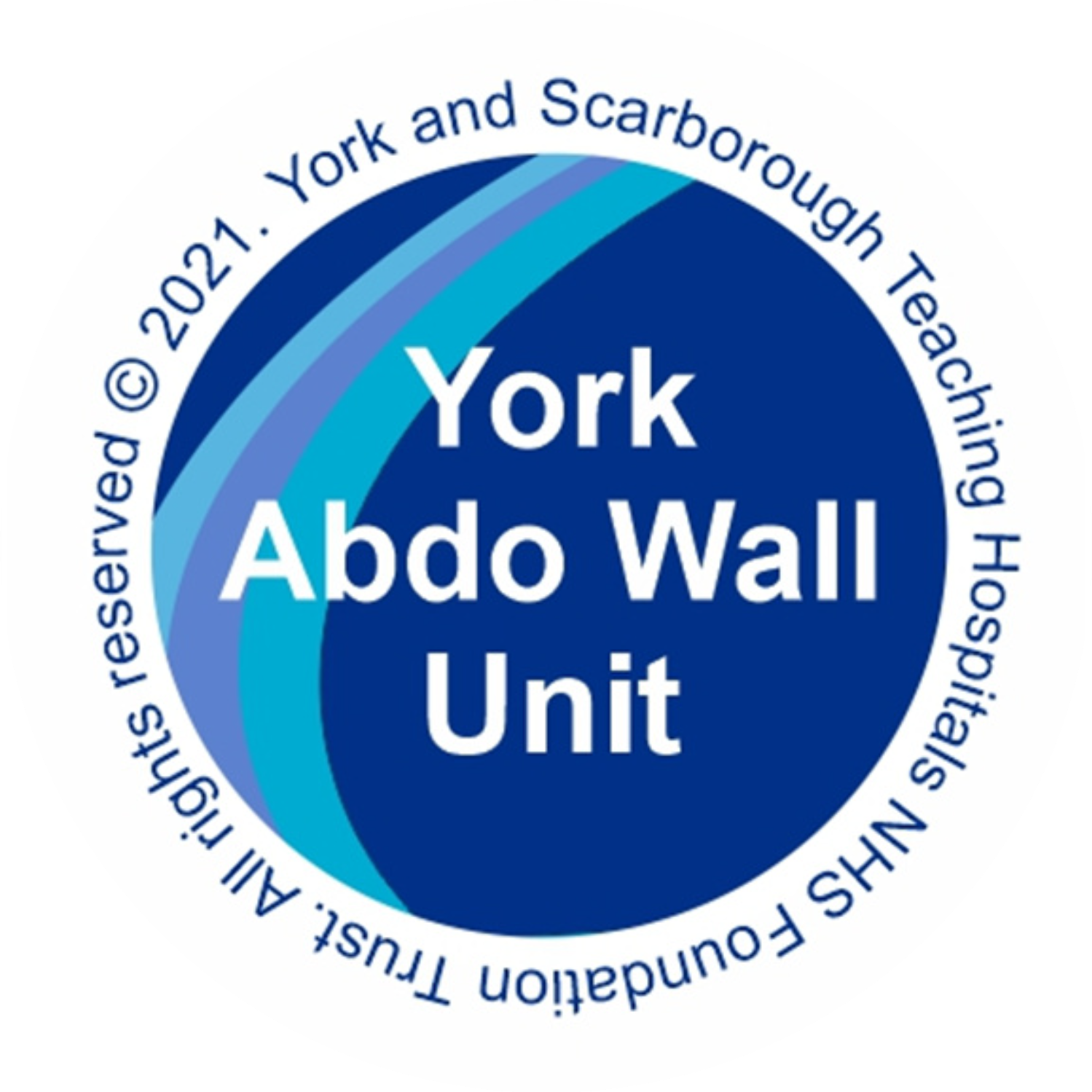If ‘yes’ please give details of any treatment you are receiving for your kidney disease? e.g. dialysis | | | | | |
| Have you ever been diagnosed as having hepatitis? | |  |  |  | |
| Do you drink more than 1.5 pints of beer or 3 shorts or a half bottle of wine per day most days? | |  |  |  | |
| Have you ever been diagnosed as having a blood clot in the leg (deep vein thrombosis) or in the lung (pulmonary embolism)? | |  |  |  | |
| Have you or any close relative, been diagnosed with an inherited blood disorder such as sickle cell disease, clotting or bleeding disorder? | |  |  |  | |
| **13. Brain, nerve & musculoskeletal disorders** | **Yes** | | **No** | | **Further details** |
| Have you been diagnosed as having epilepsy? |  | |  | |  |
| How frequent are your seizures? | | | | | |
| Do you suffer from fainting or blackouts? |  | |  | |  |
| Have you ever had a minor (TIA) or major stroke? |  | |  | |  |
| Do you have any other neurological disease such as multiple sclerosis? |  | |  | |  |
| If ‘yes’ please give details | | | | | |
| Have you been diagnosed as having arthritis? |  | |  | |  |
| Are you able to lie flat comfortably? |  | |  | |  |


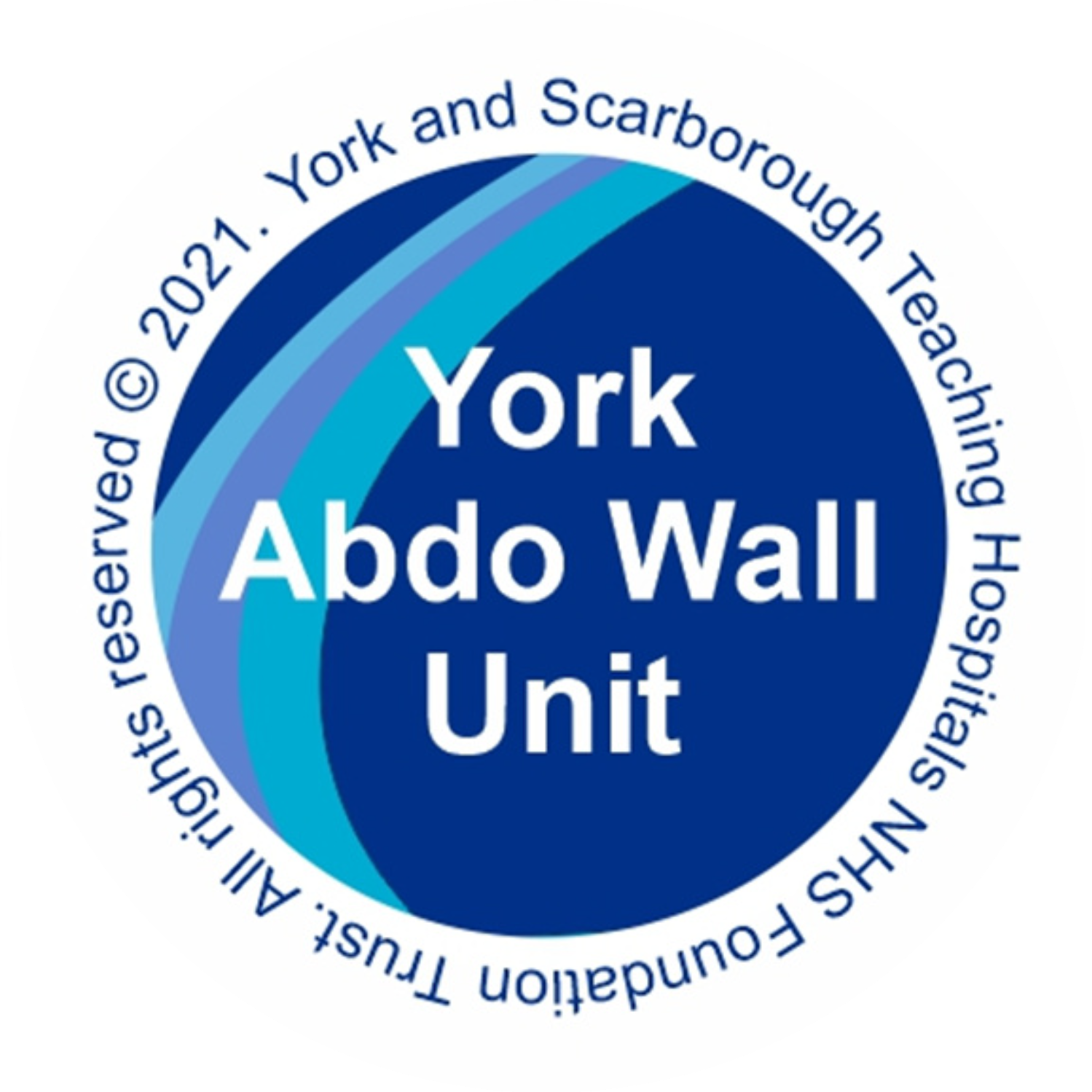


| **14. Medications** |  |  |  | |
| --- | --- | --- | --- | --- |
| Are you currently taking any medications (prescribed, herbal, over the counter, recreational, vitamins or other)?  Please give details (IN CAPITALS) or attach GP list | | | | |
| Name of medicine | Dose | | | Frequency |
| 1 |  | | |  |
| 2 |  | | |  |
| 3 |  | | |  |
| 4 |  | | |  |
| 5 |  | | |  |
| 6 |  | | |  |
| 7 |  | | |  |
| 8 |  | | |  |
| 9 |  | | |  |
| 10 |  | | |  |
| 11 |  | | |  |
| 12 |  | | |  |
| 13 |  | | |  |
| 14 |  | | | 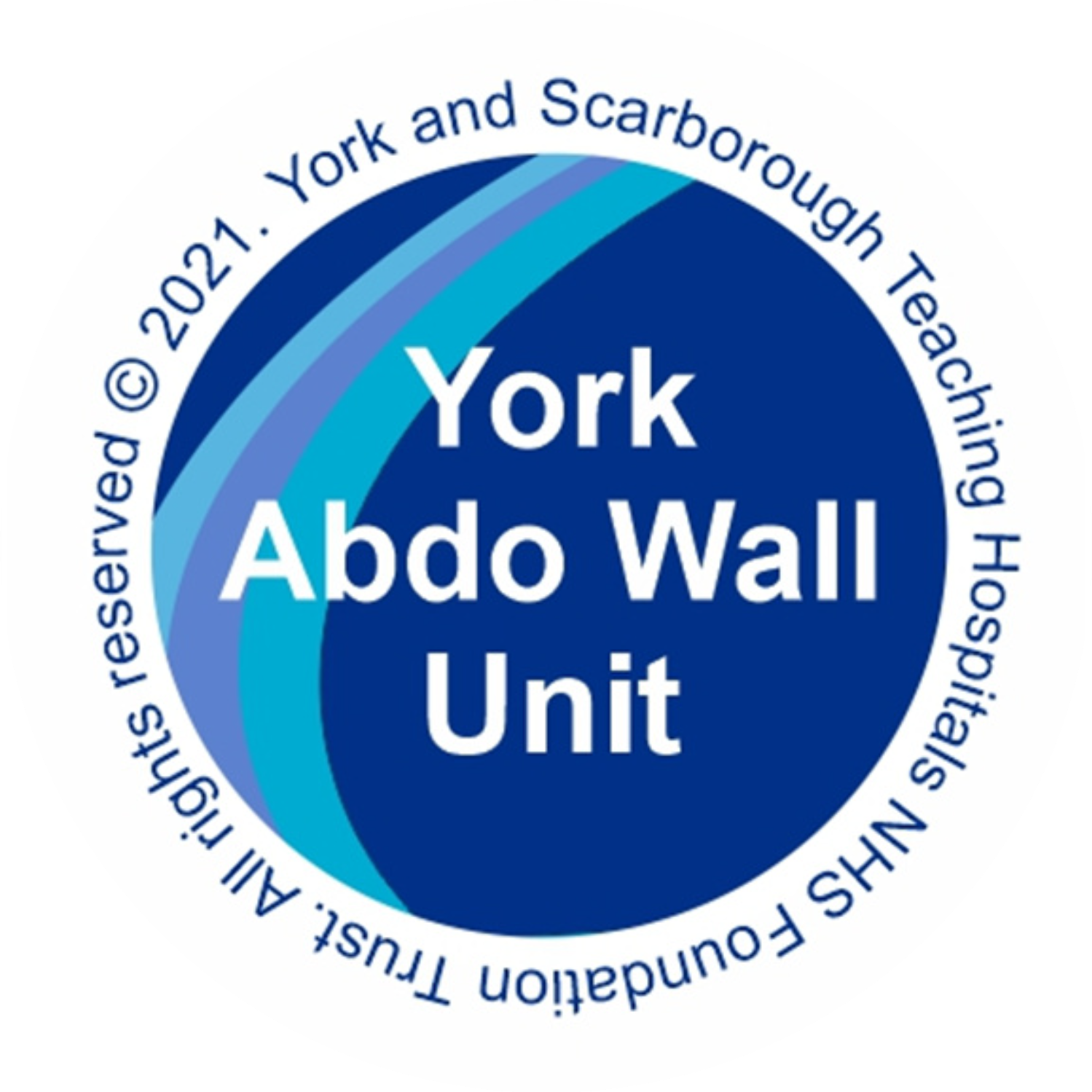 |
| 15 |  | | |  |
| 16 |  | | |  |

| **Please indicate if you are taking any of the following?** | **Yes** | **No** | **Further details** |
| --- | --- | --- | --- |
| Anticoagulant tablets (for example aspirin, dipyridamole, warfarin, clopidogrel, prasugrel, dabigatran, apixaban) |  |  |  |

| **15. Allergies** | **Yes** | **No** | **Further details** |
| --- | --- | --- | --- |
| Have you ever had a reaction to medicines or other substances (e.g. food/topical agents/latex/metal/other)? If ‘yes’ please give details. |  |  |  |

| **16. Other medical conditions** | **Yes** | **No** | **Further details** |
| --- | --- | --- | --- |
| Is there any other medical condition or problem, not previously mentioned, that you feel we should know about? |  |  | 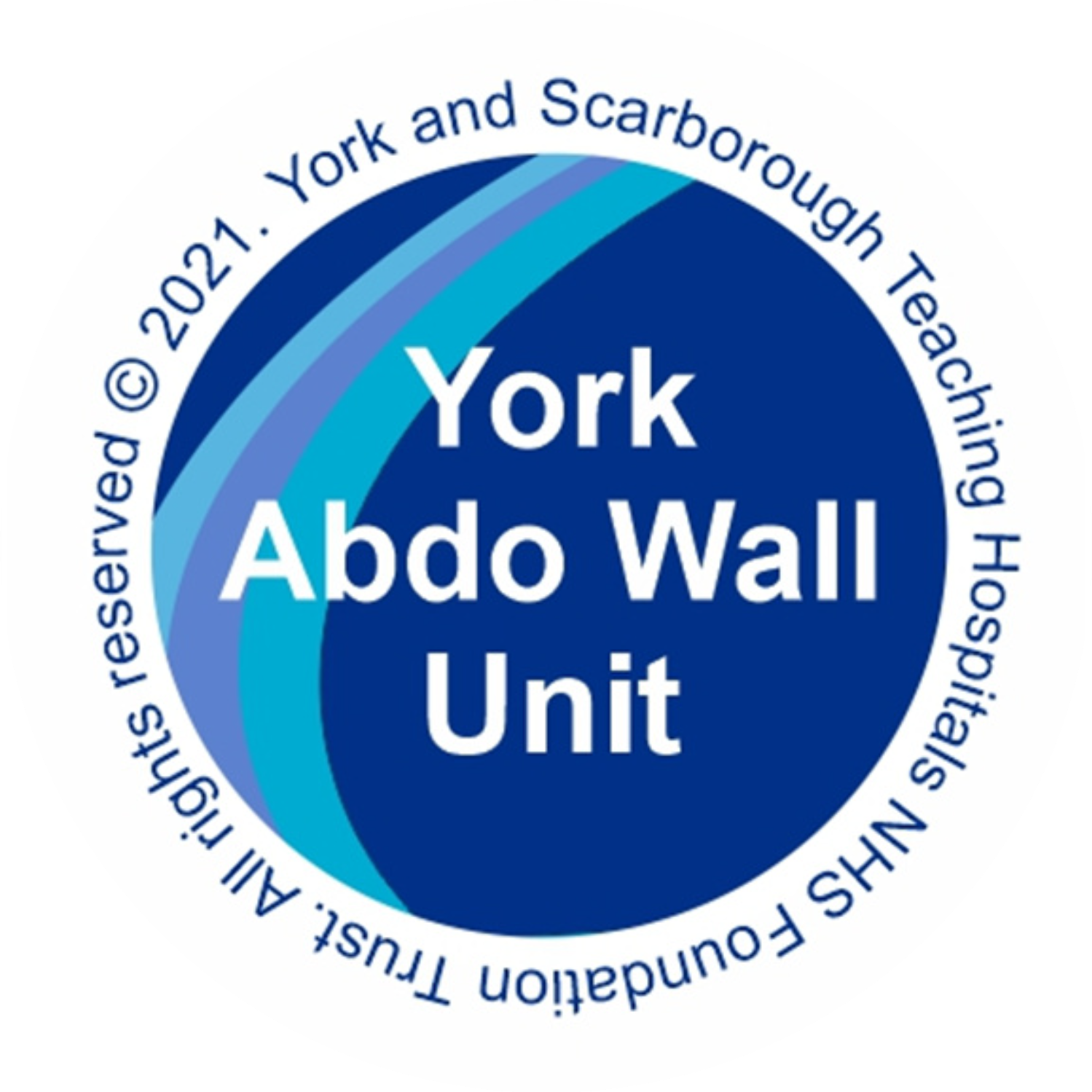 |

**In this section we would like to explore how your hernia is affecting you.**

| **17. Quality of Life** |
| --- |
| We would like you to tell us how the abdominal wall hernia affects you and your quality of life.  Our previous patients have told us the following areas they have been affected in.  You may find that some or all of these areas are applicable to you.  Please tick the area(s) you are affected by.   |  | **Yes** | **No** | | --- | --- | --- | | **Symptoms** |  |  | | **Body Image** |  |  | | **Mental Health** |  |  | | **Relationships (social and sexual)** |  |  | | **Employment** |  |  | |


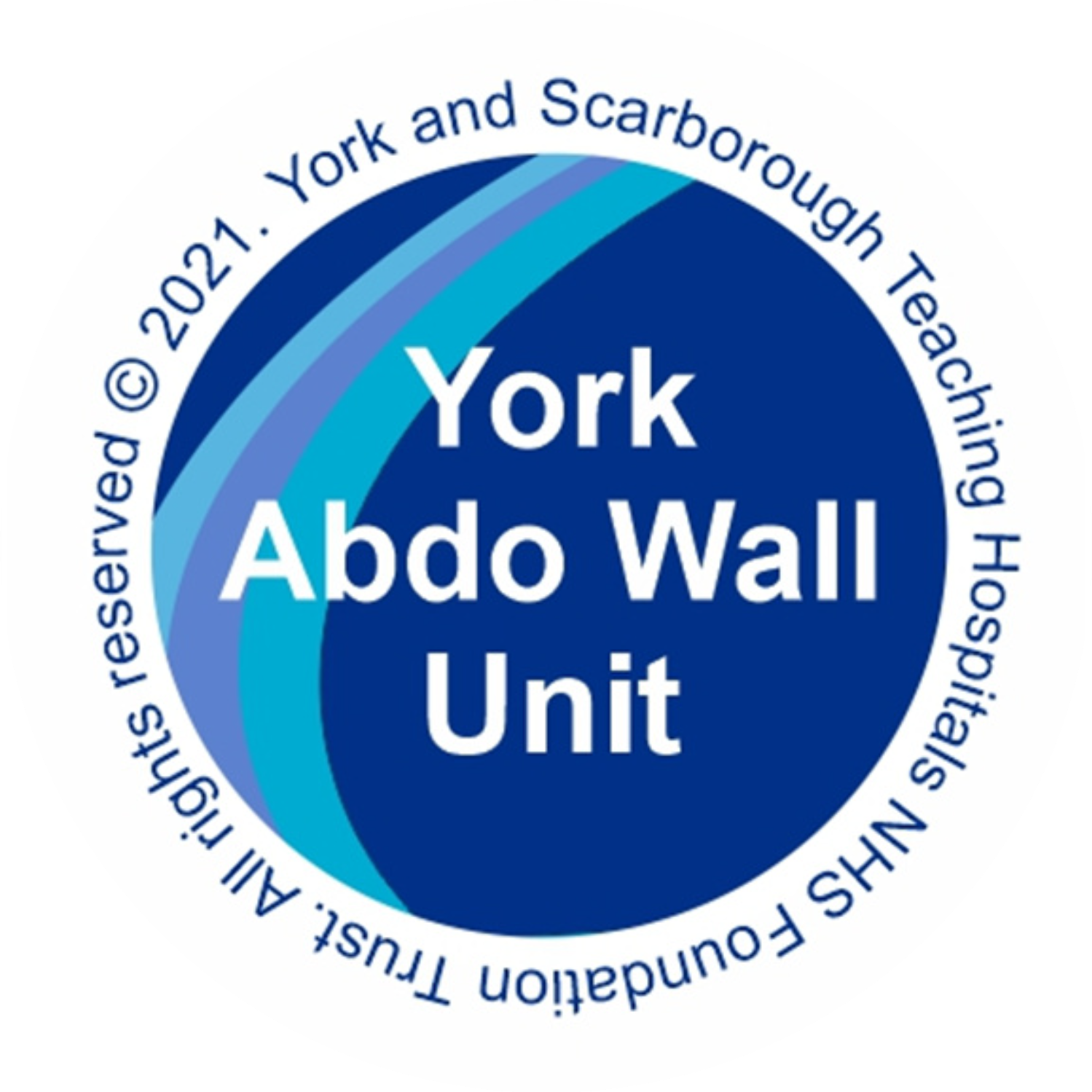


| **Symptoms:** (Restrictions and adaptations; freedom of movement; management of pain). We wish to explore what symptoms you have.   1. Does the hernia restrict your activities? 2. Have you had to adapt because of the restrictions you face? 3. Does the hernia affect the way you move (such as climbing stairs, walking for distances etc)? 4. Does the hernia cause you pain or discomfort (If so, how do you cope)?   **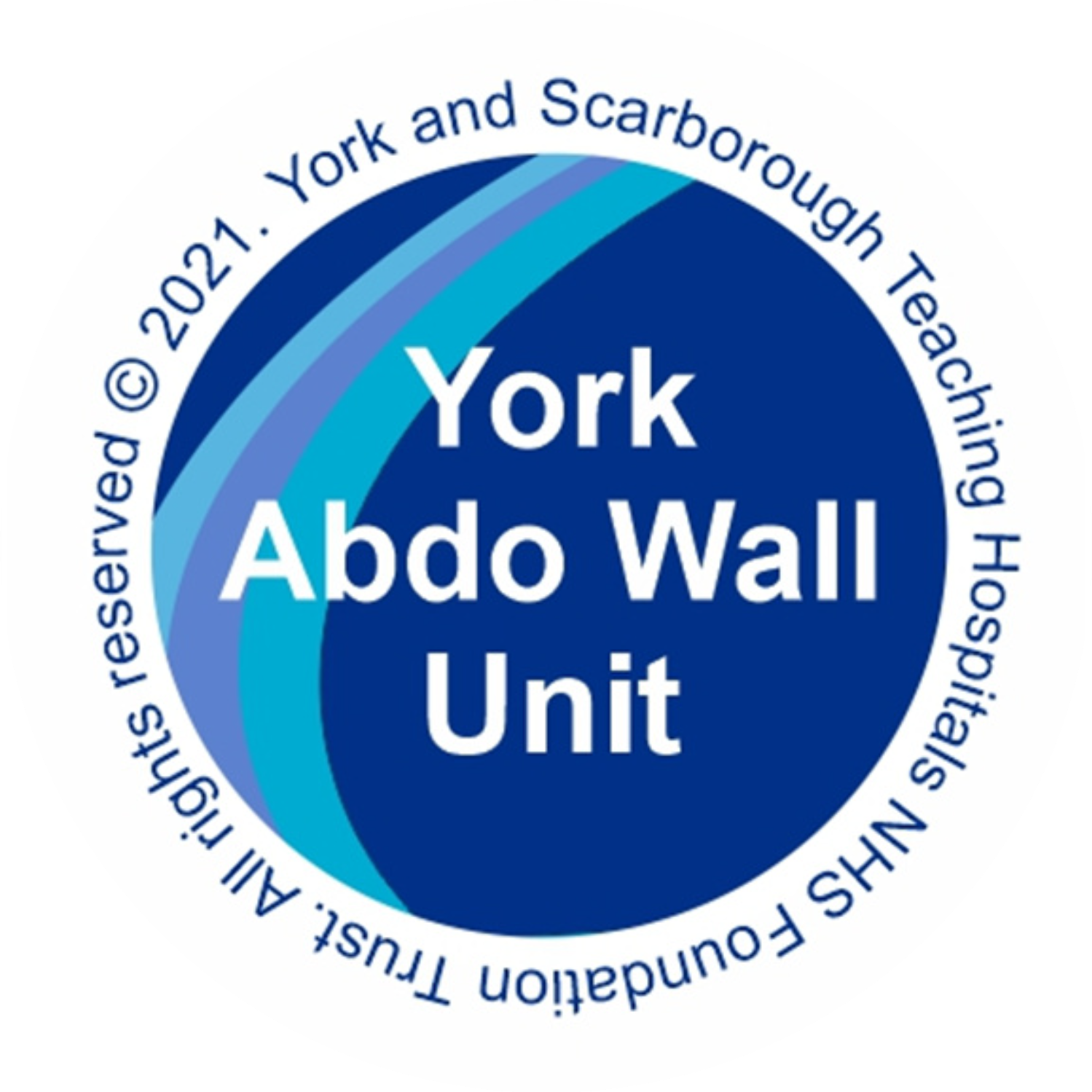** |
| --- |

| **Body Image**: (changes to perceptions of self; fears concerning perceptions of others).   1. Does the hernia make you self-conscious/embarrassed? 2. Do you feel others are noticing your hernia?   **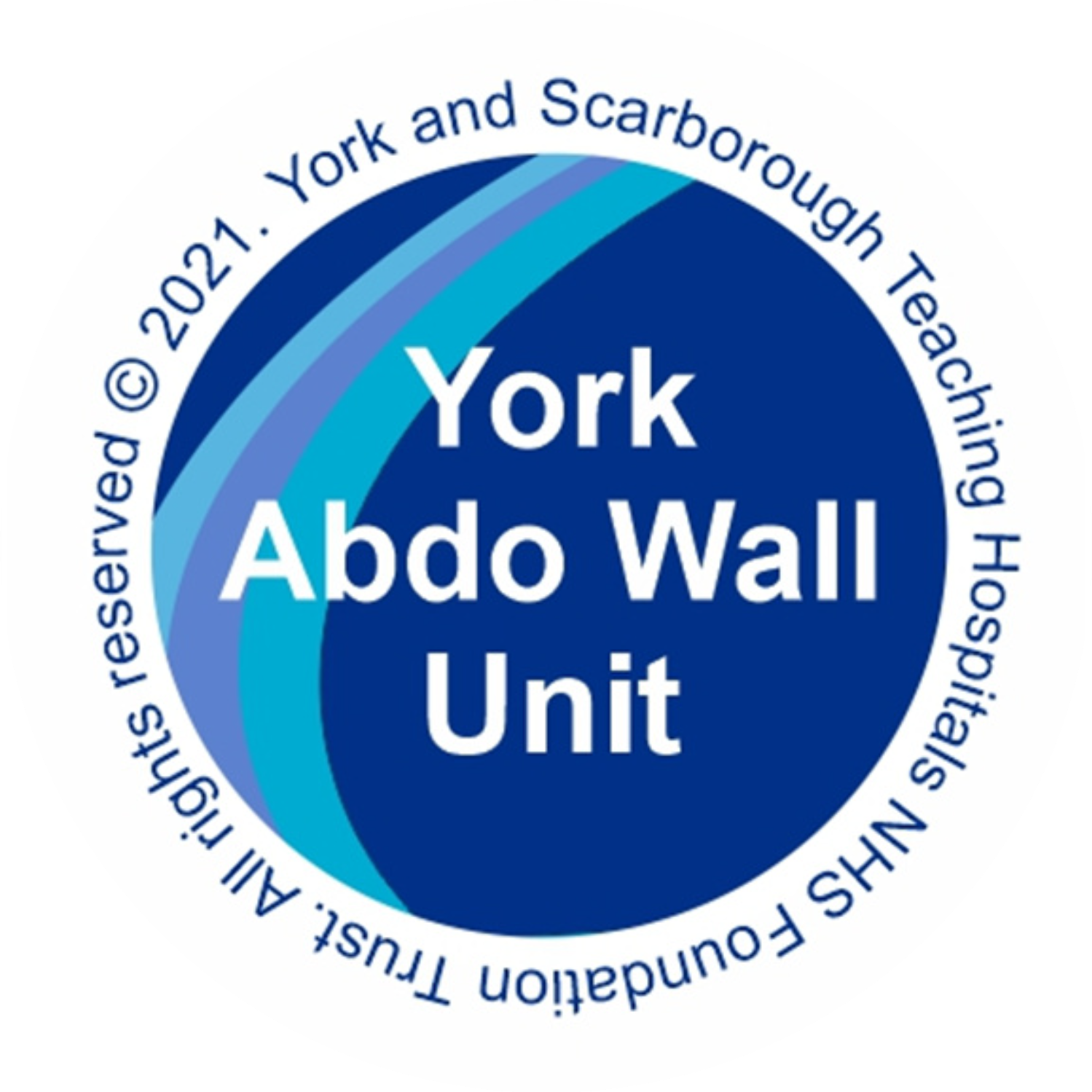** |
| --- |

| **Mental Health:** (Emotional responses; disruptions to previous identity; coping strategies).   1. Has the hernia affected you mentally (if so, please elaborate)? 2. How do you cope with this?   **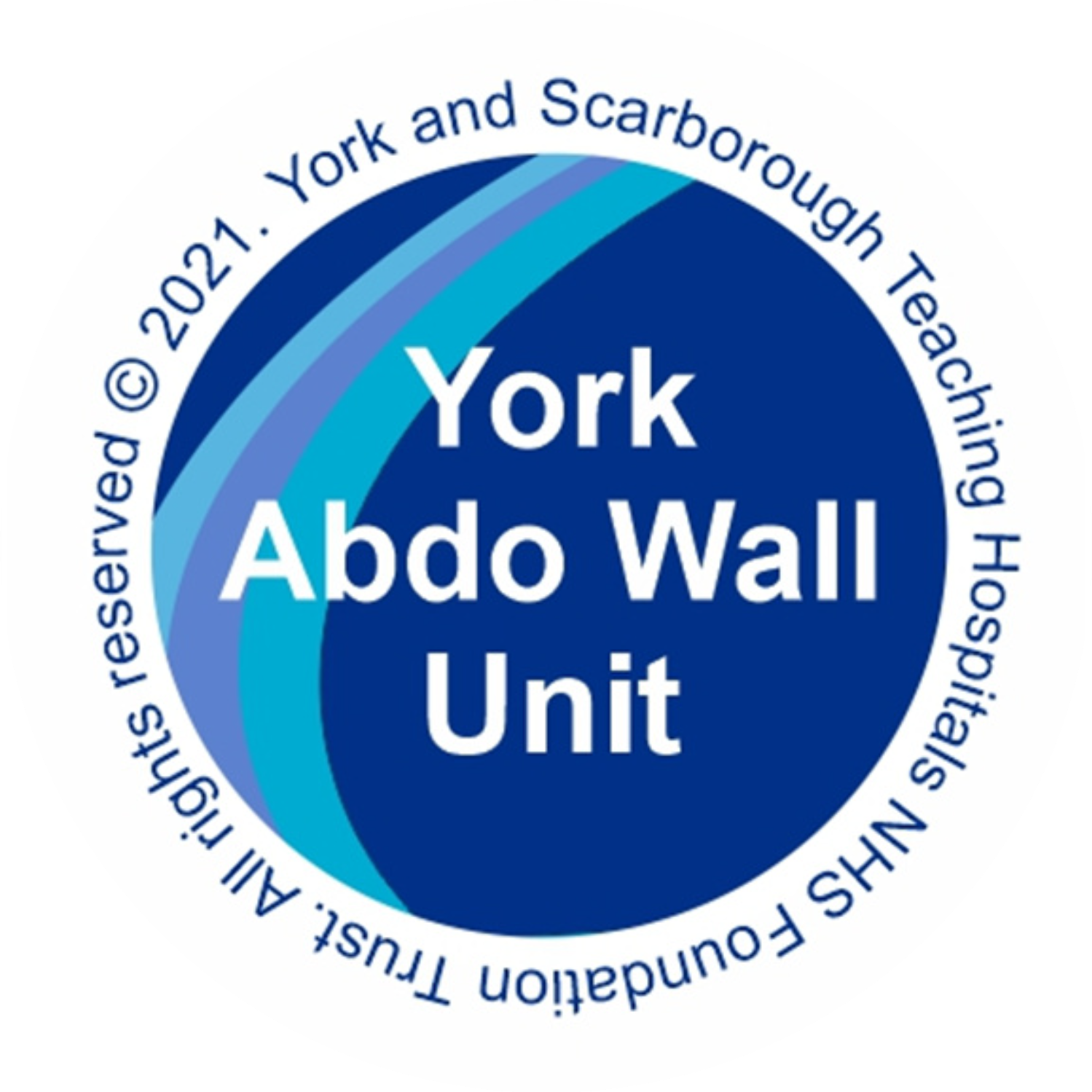** |
| --- |

| **Interpersonal relationships:** (Changes in sexual relations; difficulties in connecting socially).  For some patients the hernia affects them socially or personally.   1. Does the hernia affect you socially (if so, please elaborate)? 2. Does the hernia affect your intimate relationships (if so, please elaborate)?   **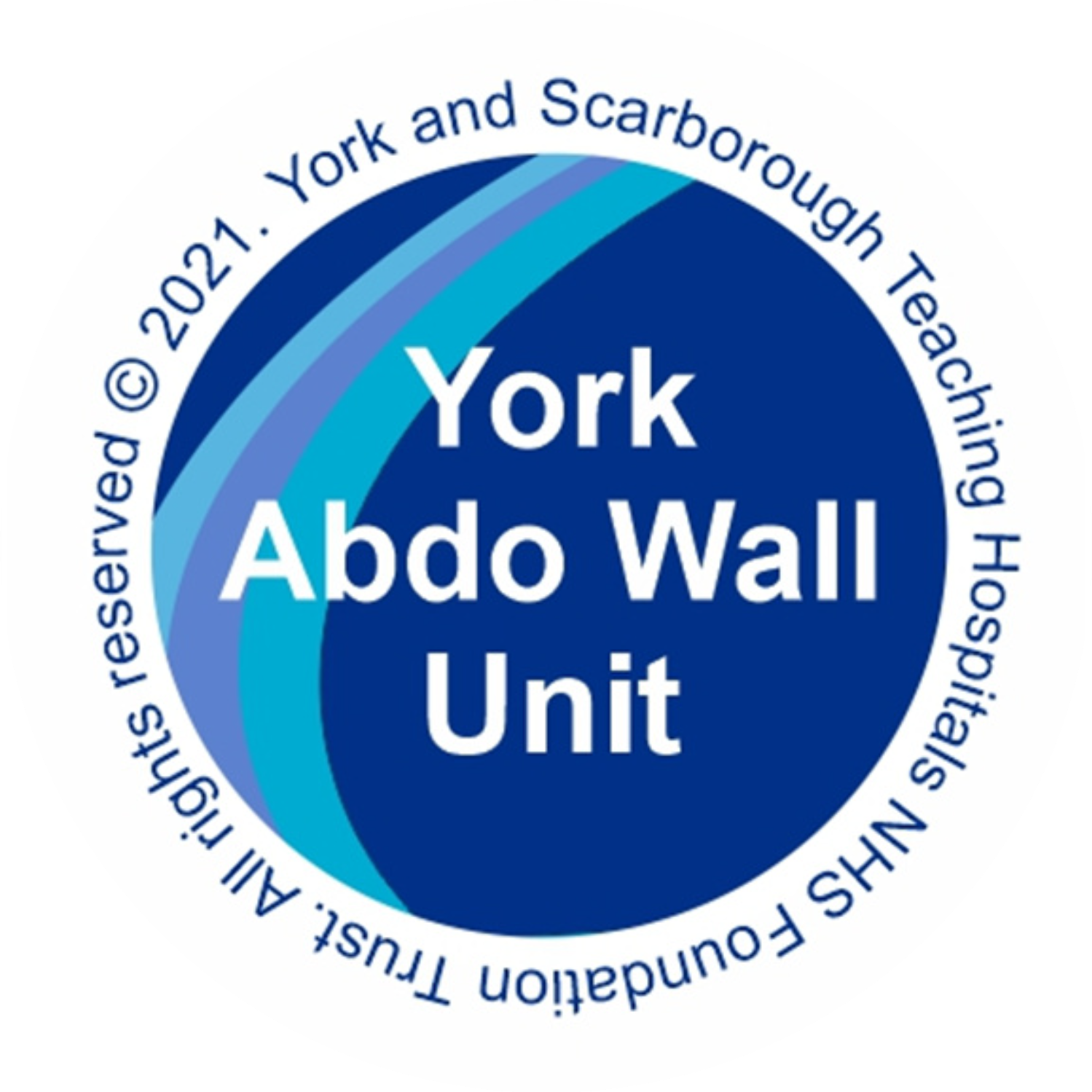** |
| --- |

| **Employment:** (Costs to family; return to work issues; financial pressure).  Are you currently working? (please circle one option)  Yes No  If yes, what do you do for a living?   1. Has the hernia affected your ability to work? 2. Have you changed your work because of the hernia? 3. Does this affect you financially?   **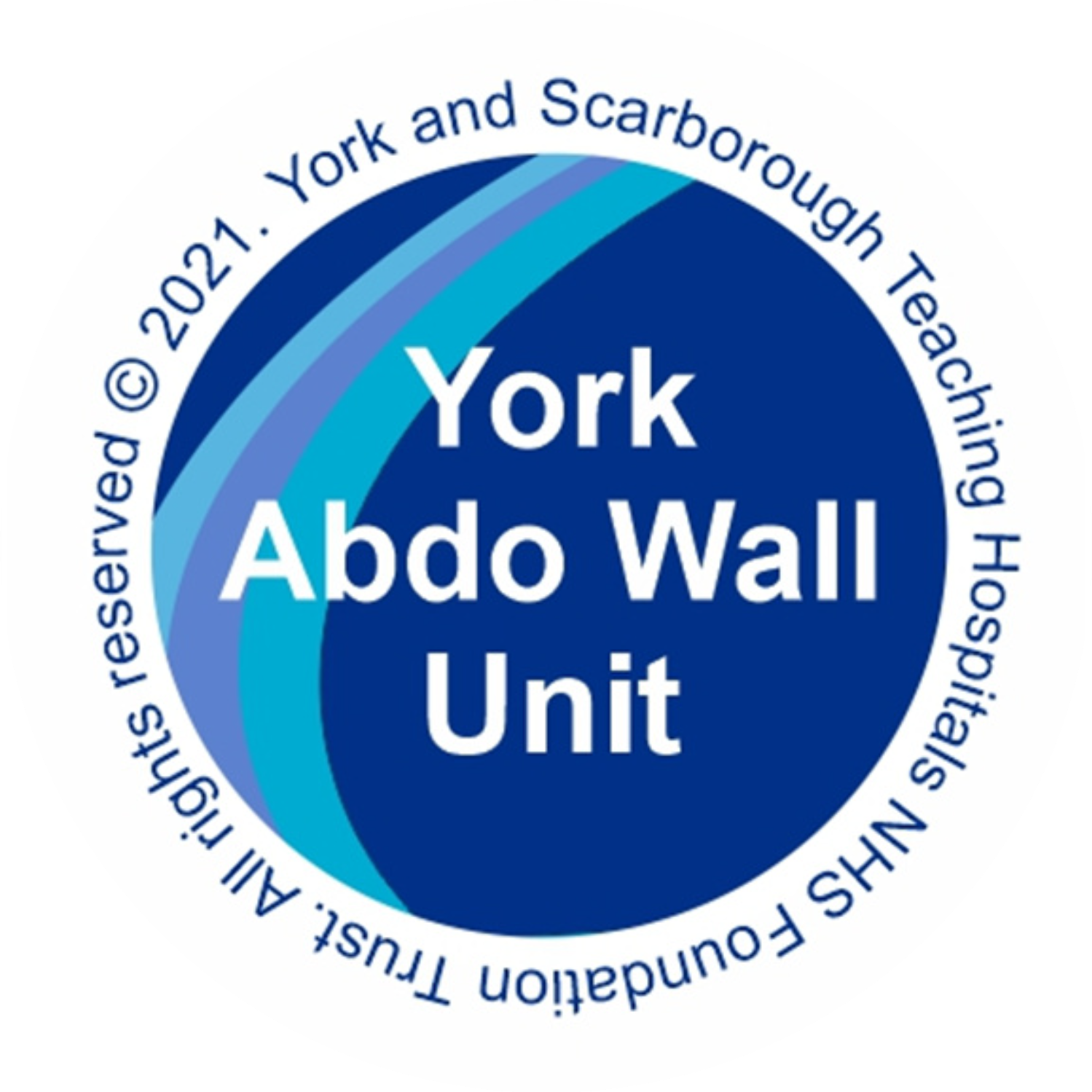** |
| --- |

| Shared decision making |
| --- |
| Please take time to answer the following questions, thinking about your answers and what you would like to achieve from the consultation.   1. What questions would you like to ask at the consultation? 2. What do you hope will happen as a result of the consultation? 3. What matters to you?       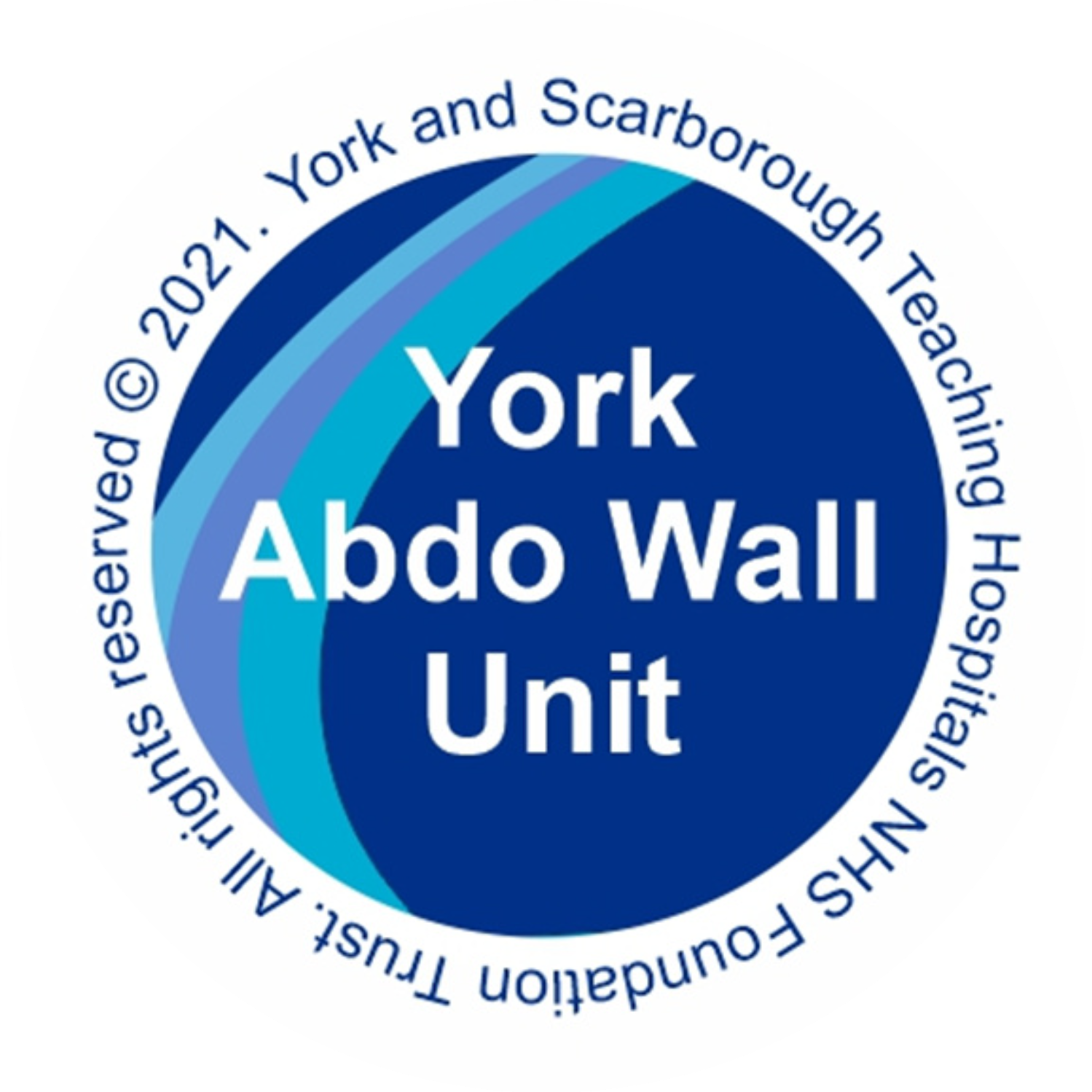 |

Clinical Photographs:

As part of the surgical planning process we normally take measurements of the hernia together with clinical photographs. Please read the enclosed information leaflet about your consent for clinical photographs and if you are in agreement please sign the enclosed consent form and bring this together with this health questionnaire to your consultation.

**NB: This consent form will cover all future photographs with regards to the treatment of your abdominal wall hernia.**

We look forward to seeing you on the day.


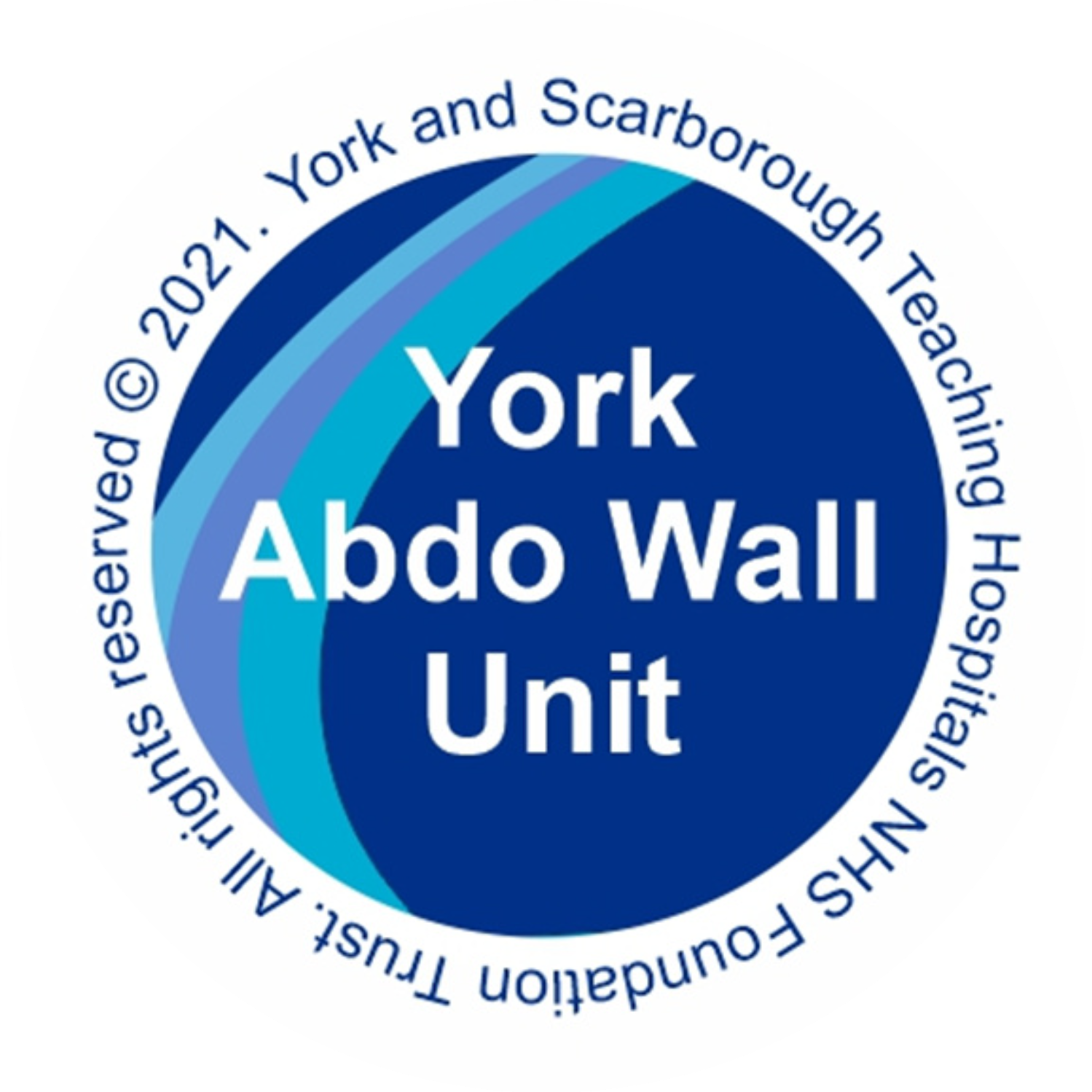


**Patient**

**Information**

**Leaflet**

**Consenting to Clinical Photography or Video recording**

Information for patients, relatives and carers

🛈
For more information, please contact your doctor or nurse

Caring with Pride

Clinical photography is used to visually record a patient’s medical condition and forms part of the patient’s clinical record.

York Teaching Hospital NHS Foundation Trust has a policy to give you the right to control the use of photographs or video recordings, which may be taken during the course of your treatment.

You can refuse to have photographs or videos taken for any reason other than for your health records. This will not affect your treatment in any way.

We will ask you to sign a consent form (reference FYCON128-3 consent to clinical photography/video) giving permission for having the photographs or videos taken. The form records that you understand what is to be done and confirm that you wish to proceed. Please make sure that you are given the opportunity to discuss any concerns and to ask any questions you may still have before signing the form. A copy of the form will be kept in your medical notes and you can have a copy for your own records.

Kindness● Openness● Excellence

You have been asked to have medical photographs or video recordings taken. These will be for:

1. Your health record - Medical photography (pre-and post-operative) is part of the clinical record. You may not be asked for your written consent for this.

Clinical photographs are taken to help doctors and other health professionals to monitor clinical conditions and plan treatments including surgery.

**If you are having Breast Surgery**

The Association of Breast Surgery (ABS) and British Association of Plastic Reconstructive and Aesthetic Surgery (BAPRAS) guidelines recommend pre and successive postoperative clinical photographs for all patients having oncoplastic and reconstructive breast surgery.

1. The teaching of health professionals and students studying healthcare here and in other hospitals/colleges/universities.
2. The education of patients with conditions similar to your own.
3. For publication in Medical and Scientific Journals or Textbooks either now or at any time in the future.

If used, for some other specific purpose, this will be explained on the consent form.

You can say yes to as many or as few of the above as you wish. Please be aware that once photographs have been published, you cannot withdraw your consent.

Kindness● Openness● Excellence

Anonymity

Every effort will be made to maintain patient anonymity/ conceal identity and patient’s faces will not be included in photographs unless specific separate consent is sought where this is unavoidable.

Storage

All digital images will be stored on a secure server with limited access or on an encrypted memory stick.

Further Information

If you have any further questions, please speak to your doctor or nurse.

Tell us what you think of this leaflet

We hope that you found this leaflet helpful. If you would like to tell us what you think, please contact Mr K Munot, Consultant Surgeon, The York Hospital, Wigginton Road, York, YO31 8HE or telephone 01904 726350.

Teaching, training and research

Our Trust is committed to teaching, training and research to support the development of health and healthcare in our community. Healthcare students may observe consultations for this purpose. You can opt out if you do not want students to observe. We may also ask you if you would like to be involved in our research.

Patient Advice and Liaison Service (PALS)

PALS offers impartial advice and assistance to patients, their relatives, friends and carers. We can listen to feedback (positive or negative), answer questions and help resolve any concerns about Trust services. PALS can be contacted on 01904 726262, or email pals@york.nhs.uk. An answer phone is available out of hours.

Providing care together in York, Scarborough, Bridlington,
Malton, Selby and Easingwold communities


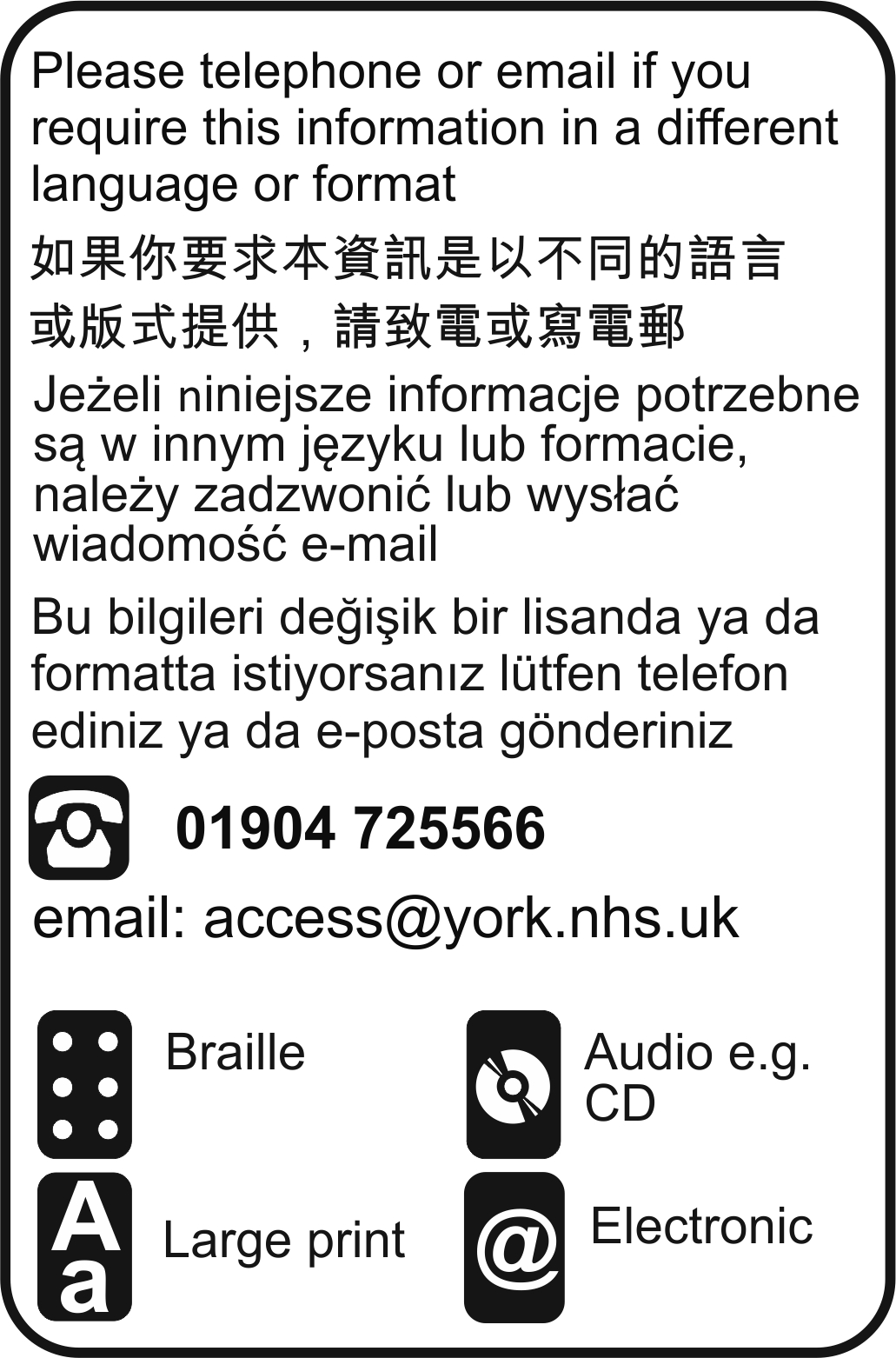


| Owner |  | |
| --- | --- | --- |
| Date first issued |  |  |
| Review Date |  |  |
| Version | 1 (reissued June 2017) |  |
| Approved by | Breast Surgeons and Surgery |  |
| Linked to consent form | FYCON128-3 consent to photography/video v1 |  |
| Document Reference | PIL 888 v1 |  |

© 2017 York and Scarborough Teaching Hospitals NHS Foundation Trust.

All Rights reserved.

[www.yorkhospitals.nhs.uk](http://www.yorkhospitals.nhs.uk/)

To be completed by the Consultant:

Patient Label

**Abdominal Wall Reconstruction - Photography**

Date: …………………..

- Please take Abdominal wall photograph as per agreed protocol
- Consent for medical photography has been obtained
- I request the photographs are uploaded to the folder

Consultant Signature…………………………………………………………………..


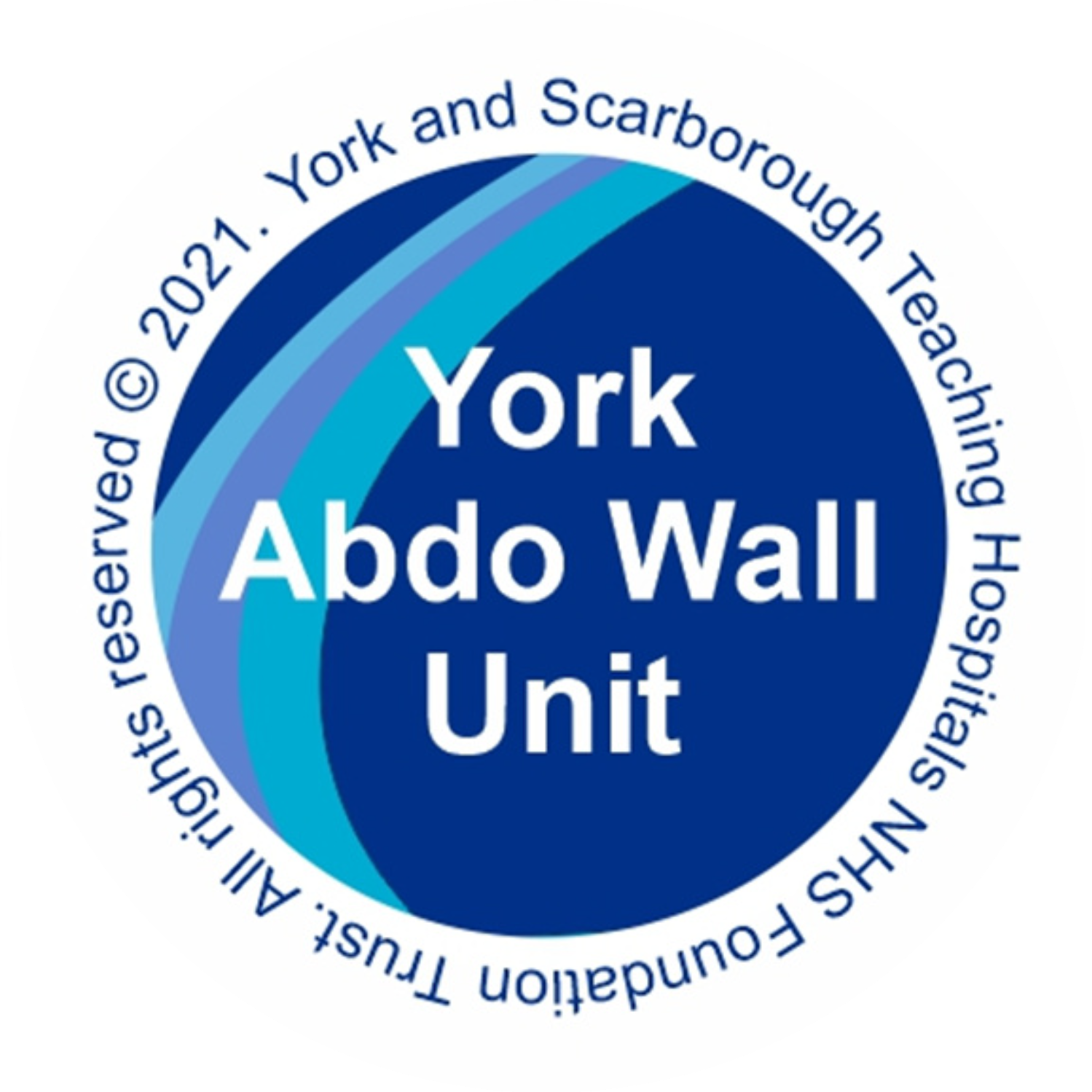

Supplement: Supplementary file 5 — Supplementary Material 5 (DOCX 2.68 MB) [file 10029_2025_3489_MOESM5_ESM.docx]
